# Genetic Causes of Phenotypic Adaptation to the Second Fermentation of Sparkling Wines in *Saccharomyces cerevisiae*

**DOI:** 10.1534/g3.116.037283

**Published:** 2016-11-28

**Authors:** Maria Martí-Raga, Emilien Peltier, Albert Mas, Gemma Beltran, Philippe Marullo

**Affiliations:** * Departament de Bioquímica i Biotecnologia, Facultat d’Enologia, Universitat Rovira i Virgili, 43007 Tarragona, Spain; † Unité de recherche OEnologie, EA 4577, ISVV, Université Bordeaux, 33882 Villenave d’Ornon, France; ‡ Biolaffort, 33100 Bordeaux, France

**Keywords:** heterosis, *PMA1*, *VMA13*, *MSB2*, *PDR1*

## Abstract

Hybridization is known to improve complex traits due to heterosis and phenotypic robustness. However, these phenomena have been rarely explained at the molecular level. Here, the genetic determinism of *Saccharomyces cerevisiae* fermentation performance was investigated using a QTL mapping approach on an F1-progeny population. Three main QTL were detected, with positive alleles coming from both parental strains. The heterosis effect found in the hybrid was partially explained by three loci showing pseudooverdominance and dominance effects. The molecular dissection of those QTL revealed that the adaptation to second fermentation is related to pH, lipid, or osmotic regulation. Our results suggest that the stressful conditions of second fermentation have driven the selection of rare genetic variants adapted to maintain yeast cell homeostasis and, in particular, to low pH conditions.

In higher eukaryotes, hybridization is known to improve fitness and complex traits (Crow 1998; Lippman and Zamir 2007), accelerate evolution (Grant and Grant 1992), and confer better adaptation in novel or changing environments (Zeyl and Bell 1997). The benefits of hybridization are mainly due to heterosis (hybrid vigor) and homeostasis (robustness to environmental changes). In organisms of agronomical interest, such phenomena are widely documented (Fridman 2014; Lippman and Zamir 2007) and have been used for decades in plant and animal breeding programs (Welcker *et al.* 2005; Cassady *et al.* 2002; Crow 1998).

Intra- and interspecific hybridization also plays a major role in phenotypic adaptation and evolution in fungi and yeasts. At the genomic scale, many comparative studies have shown that polyploidization (Albertin and Marullo 2012; Borneman *et al.* 2012; Curtin *et al.* 2012), reticulated evolution (Novo *et al.* 2009; Almeida *et al.* 2014), or interspecific hybridization (Morales and Dujon 2012) strongly drive the evolution of these eukaryotic microbes. Over the past 5 yr, *Saccharomyces* yeasts have emerged as model organisms to study hybrid vigor and homeostasis by comparing hybrids and their relative parents, both at the intra- (Zörgö *et al.* 2012; Timberlake *et al.* 2011; Shapira *et al.* 2014) and interspecific (da Silva *et al.* 2015; Dunn *et al.* 2013; Stelkens and Brockhurst 2014) level. The study of phenotypic responses of hybrids in various environmental conditions has provided a broad overview of hybridization benefits, highlighting phenotypic novelty (da Silva *et al.* 2015), heterosis (Timberlake *et al.* 2011; Zörgö *et al.* 2012; Shapira *et al.* 2014; da Silva *et al.* 2015), outbreeding transgression (Stelkens *et al.* 2014), and homeostasis (da Silva *et al.* 2015). However, the underlying genetic determinisms of these benefits have yet to be elucidated.

Chromosomal regions linked to heterosis (Semel *et al.* 2006) and homeostasis (Fraser *et al.* 2005; Bhatia *et al.* 2014) have, with great difficulty, been identified using quantitative genetic approaches in higher eukaryotes. However, such QTL have rarely been dissected at the gene level (Krieger *et al.* 2010). Nowadays, thanks to its powerful genetics and its small genome, the budding yeast (*Saccharomyces cerevisiae*) has emerged as a promising model to achieve this task (Liti and Louis 2012). In 2002, a pioneering work identified one heterotic QTL (Steinmetz *et al.* 2002) resulting from the combined effect of pseudooverdominance (Sinha *et al.* 2006) and epistasis (Sinha *et al.* 2008). Although this organism has been used for many other QTL mapping studies (Zimmer *et al.* 2014; Yang *et al.* 2013; Marullo *et al.* 2007a; Ambroset *et al.* 2011; Brice *et al.* 2014; Hubmann *et al.* 2013; Cubillos *et al.* 2011; Jara *et al.* 2014; Gutiérrez *et al.* 2013), further molecular examples of heterosis and homeostasis effects are still needed.

*S. cerevisiae* plays a crucial role in the production of food, beverages, biofuels, and biochemicals. Therefore, QTL mapping has been employed to identify natural genetic variations in various industrial contexts, such as biofuel (Swinnen *et al.* 2012; Hubmann *et al.* 2013) and wine (Marullo *et al.* 2007a; Ambroset *et al.* 2011; Gutiérrez *et al.* 2013; Jara *et al.* 2014; Zimmer *et al.* 2014).

The harsh physiological conditions found in these industrial processes (low pH, high ethanol content, extreme temperature, and low nitrogen availability) promote the efficacy of natural selection (Goddard *et al.* 2005; Zeyl 2006), creating favorable conditions for the emergence of particularly well-adapted strains. In this study, we investigated the behavior of *S. cerevisiae* wine strains during the production of sparkling wines such as Champagne and Cava. This particular winemaking process consists of two consecutive fermentations. A primary fermentation is conducted to obtain a base wine from grape must. The obtained base wine is then mixed with sugar and yeast to achieve a second fermentation stage that occurs inside the sealed bottle (Carrascosa *et al.* 2011). Due to the extreme conditions (low pH, high ethanol, and a steady increase in CO_2_ pressure), several weeks are required to complete this second fermentation (Ribéreau-Gayon *et al.* 2006). We recently found that the second fermentation kinetics are significantly affected by the choice of the yeast strain with which the fermentation is conducted (Martí-Raga *et al.* 2015). In order to decipher the molecular basis of this phenotypic discrepancy, we applied a QTL mapping approach using NGS-based genotyping. Four genes involved in the genetic determinism of second fermentation kinetics were identified. These genes play a central role in maintaining cell homeostasis, such as intracellular pH regulation, yeast cell detoxification, control of plasma membrane composition, and the response to cold stress. Furthermore, we can formulate a plausible molecular explanation for the observed heterosis and identified genetics × environment interactions explaining the phenotypic robustness of the hybrid to pH variations.

## Materials and Methods

### Yeast strains used and culture conditions

All of the *S. cerevisiae* strains used are listed in Table 1. Both parental strains are monosporic clones derived from wine yeast starters. They were both used under their diploid (GN and SB) and haploid (hoGN and hoSB) forms. A set of 117 haploid segregants of the hybrid BN (hoSB × GN) was obtained by tetrad microdissection, as described in Marullo *et al.* (2006), and was used for QTL mapping. All strains were grown at 28° on YPD medium (1% yeast extract, 1% peptone, and 2% glucose), solidified with 2% agar when required. When necessary, the antibiotic G418 (Sigma-Aldrich, St Louis, MS) and Nourceothricin (Werner bioagent, Germany) was added to the media at a final concentration of 100 μg/ml. Sporulation was induced on ACK medium (1% potassium acetate and 2% agar) during 3 d at 24°.

**Table 1:** Yeast strains used in the study

Strain	Genetic Background	Genotype*^a^*^,^*^b^*^,^*^c^*^,^*^d^*^,^*^e^*	Origin*^f^*
Y04381	S288c	BY4741; *mat a*; *his3*Δ*1*; *leu2* Δ *0*; *met15* Δ *0*; *ura3* Δ *0*; *YGL013c*::*kanMX4*	Euroscarf
Y06979	S288c	BY4741; *mat a*; *his3*Δ*1*; *leu2* Δ *0*; *met15* Δ *0*; *ura3* Δ *0*; *YGR032w*::*kanMX4*	Euroscarf
Y05455	S288c	BY4741; *mat a*; *his3*Δ*1*; *leu2* Δ *0*; *met15* Δ *0*; *ura3* Δ *0*; *YPR036w*::*kanMX4*	Euroscarf
Y06978	S288c	BY4741; *mat a*; *his3*Δ*1*; *leu2* Δ *0*; *met15* Δ *0*; *ura3* Δ *0*; *YGR028w*::*kanMX4*	Euroscarf
Y04644	S288c	BY4741; *mat a*; *his3*Δ*1*; *leu2* Δ *0*; *met15* Δ *0*; *ura3* Δ *0*; *YGR014w*::*kanMX4*	Euroscarf
Y05451	S288c	BY4741; *mat a*; *his3*Δ*1*; *leu2* Δ *0*; *met15* Δ *0*; *ura3* Δ *0*; *YPR032w*::*kanMX4*	Euroscarf
Y24376	S288c	BY4743; *mat a/* α ; *his3*Δ*1/his3* Δ *1*; *leu2* Δ *0/leu2* Δ *0*; *lys2* Δ *0/LYS2*; *MET15/met15* Δ *0*; *ura3* Δ *0/ura3* Δ *0*; *YGL008c*::*kanMX4/YGL008c*	Euroscarf
Y24639	S288c	BY4743; *mat a/* α ; *his3*Δ*1/his3* Δ *1*; *leu2* Δ *0/leu2* Δ *0*; *lys2* Δ *0/LYS2*; *MET15/met15* Δ *0*; *ura3* Δ *0/ura3* Δ *0*; *YGL009c*::*kanMX4/YGL009c*	Euroscarf
GN	Monosporic clone of VL1	*HO/HO*; *chr: VIII*; *chr: XV-t-XVI*;	Marullo *et al.* (2007a)
SB	Monosporic clone of BO213	*HO/HO*, *chr:VIII*, *chrXV*, *chr:XVI*	Marullo *et al.* (2007a)
hoGN	GN	Haploid derivate of GN, *ho*::*NATMX4*, *mat a*	Albertin *et al.* (2013)
hoSB	SB	Haploid derivate of SB, *ho*::*kanMX4*, *mat* α	Albertin *et al.* (2013)
BN	F1 hybrid	hoSB×GN hybrid, *ho*::*kanMx4/HO*, *mat a/mat* α	Marullo *et al.* (2006)
HO-BN	F1 hybrid	SB×GN hybrid, *HO/HO mat a/mat* α	Marullo *et al.* (2007b)
GΔS-PDR1	HO-BN	Hemizygote hybrid *YGL013^GN^*::*kanMX4/YGL013^SB^*	This study
SΔG-PDR1	HO-BN	Hemizygote hybrid *YGL013^GN^/YGL013^SB^*::*kanMX4*	This study
GΔS-GSC2	HO-BN	Hemizygote hybrid *YGR032^GN^*::*kanMX4/YGR032^SB^*	This study
SΔG-GSC2	HO-BN	Hemizygote hybrid *YGR032^GN^/YGR032^SB^*::*kanMX4*	This study
GΔS-VMA13	HO-BN	Hemizygote hybrid *YPR036^GN^*::*kanMX4/YPR036^SB^*	This study
SΔG-VMA13	HO-BN	Hemizygote hybrid *YPR036^GN^/YPR036^SB^*::*kanMX4*	This study
GΔS-MSP1	HO-BN	Hemizygote hybrid *YGR028^GN^*::*kanMX4/YGR028^SB^*	This study
SΔG-MSP1	HO-BN	Hemizygote hybrid *YPR028^GN^/YPR028^SB^*::*kanMX4*	This study
GΔS-MSB2	HO-BN	Hemizygote hybrid *YGR014^GN^*::*kanMX4/YGR014^SB^*	This study
SΔG-MSB2	HO-BN	Hemizygote hybrid *YGR014^GN^/YGR014^SB^*::*kanMX4*	This study
GΔS-SRO7	HO-BN	Hemizygote hybrid *YPR032^GN^*::*kanMX4/YPR032^SB^*	This study
SΔG-SRO7	HO-BN	Hemizygote hybrid *YPR032^GN^/YPR032^SB^*::*kanMX4*	This study
GΔS-PMA1	HO-BN	Hemizygote hybrid *YGL008^GN^*::*kanMX4/YGL008^SB^*	This study
SΔG-PMA1	HO-BN	Hemizygote hybrid *YGL008^GN^/YGL008^SB^*::*kanMX4*	This study
GΔS-SEC9	HO-BN	Hemizygote hybrid *YGR009^GN^*::*kanMX4/YGR009^SB^*	This study
SΔG-SEC9	HO-BN	Hemizygote hybrid *YGR009^GN^*/*YGR009^SB^*::*kanMX4*	This study

a mat a or mat α refers to the mating type of the haploid line.

b his3Δ1; leu2Δ0; met15Δ0; ura3Δ0 refers to auxotrophic markers.

c ho and HO refers to the hetero/homothalism status of the strains.

d chr: XV-t-XVI refers to the translocated form of chromosome XVI described in Zimmer *et al.* (2016).

e NATMX4 and kanMx4 refers to the antibiotic cassettes used for gene disruption.

f Euroscarf collection web site: http://www.euroscarf.de.

### Second fermentation phenotypic analysis

The strains were phenotyped for their fermentative behavior during the second fermentation according to the procedure described in Martí-Raga *et al.* (2015). Briefly, yeast cells were acclimatized before their inoculation into the base wine by successive cultures in YNB media and synthetic wine with increasing ethanol concentrations. Upon acclimatization, yeast cells were inoculated into base wine supplemented with sucrose (22 g/L) and bentonite (30 mg/L) at 0.2 units of OD_600_ (2 × 10^6^ cells/ml). The base wine used was kindly donated by Juvé & Camps (pH 3.1, ethanol concentration 9.4 g/L, and YAN content of 23.17 mg of Nitrogen per Liter). When required, the pH of the base wine was modified using phosphoric acid (85%) or sodium hydroxide (10 M). The mixture was introduced into bottles (750 ml) that were hermetically sealed. The second fermentation took place at 16°, and the fermentation performance was assessed by monitoring CO_2_ production inside the bottle though time using an aphrometer (L.sensor.CO2, L PRO SRL, Camisano Vicentino, Italy). This technique enables the measurement of pressure inside the bottle in a noninvasive way. The values were normalized according to the temperature using Henry’s law constant, and expressed as pressure at 10°. Fermentation kinetics data were fitted using the 5PL model (Gottschalk) to extract relevant parameters, such maximum pressure achieved (Pmax), fermentative rate (rate) or time to achieve 0.5, 2, and 5 bars during the fermentation (t0.5, t2, and t5, respectively) (Martí-Raga *et al.* 2015). The trait heritability and the percentage of transgression were calculated as described in Marullo *et al.* (2006).

### Genotyping and marker map construction

The whole-genome sequences of the parental strains (SB and GN) have been previously obtained by paired-end Illumina sequencing. The list of single nucleotide polymorphisms (SNPs) compared to the reference genome was extracted using the SAMtools package (Zimmer *et al.* 2014). The noncommon SNPs between parental strains were subtracted using a custom R script (Supplemental Material, File S1).

All the 117 progeny clones were genotyped by whole-genome sequencing at a low coverage (3–6 ×). Total DNA was extracted using a Wizard Genomic DNA Purification kit (Promega, Madison, WI) following the manufacturer’s instructions. DNA libraries were constructed using the Illumina Nextera XT kit (Illumina, CA) as indicated by the manufacturer. DNA libraries were then pooled and sequenced with a MiSeq apparatus using the standard kit v2 (Illumina) generating paired-end reads of 2 × 250 bp in the Université Bordeaux’s genomics facility. All sequencing data (filtering and mapping) was performed using the available tools at the public Galaxy server (https://usegalaxy.org). Sequencing data were treated as single reads. To optimize downstream analysis, quality control was applied using FASTX-Toolkit (http://hannonlab.cshl.edu/fastx_toolkit/) for every read as follows: reads were trimmed at their 3′-end to eliminate the bases with low sequencing quality, then only the reads with a Phred quality score < 20 were retained. Filtered reads were then mapped to the reference genome of *S. cerevisiae* strain s288c, using *BWA* software (Li and Durbin 2010) with default parameters. Once the reads had been mapped, BAM files were extracted and a pileup dataset was generated using SAMTools’ (Li *et al.* 2009) for every sequenced segregant. The pileup dataset was opened in R and the genotype of each segregant at the position of the noncommon SNP of the parental strains was evaluated using an R script (File S1). To construct the marker map, we retained the markers that met the following requests: having a 1:1 segregation among the progeny (Chi-χ test, > 0.05), having an even distribution along the genome (1 marker ∼15 kb), and having a genotype of ≤ 50% of the progenies. The genetic and phenotypic dataset is listed in Table S1.

### Linkage analysis

QTL mapping was performed using an R script applying a nonparametric test (Wilcoxon), in order to avoid any normality issues for every phenotype at every marker position (File S1). The linkage result (LK) was expressed as the –log_10_ of the *p*-value. To calculate the significance of a QTL, we permuted the phenotypic value 1000 times, recording the highest LK score at each permutation. We considered a QTL significant if its LK score was higher than the 0.05 tail of the 1000 permuted LK scores.

The genetic effects of the mapped QTL were then determined by an ANOVA. The ANOVA was applied following the general linear model:

$$Y_{i}=m+QTL1_{i}+QTL2_{i}+QTL3_{i}+{QTL1_{i}}^{*}QTL2_{i}+QTL2_{i}*QTL3_{i}+QTL1_{i}*QTL3_{i}+E_{i}$$

where *Y_i_* was the value of the trait according to the genotype I; *m* was the overall mean; *QTL1*, *QTL2*, and *QTL3* were the simple QTL effects; QTL1*QTL2, QTL2*QTL3, and QTL1*QTL3 were the interaction effects between QTL; and *E* the residual error. The conditions of ANOVA application were controlled by verifying the homoscedasticity (Levene test) and normal distribution of residues (Shapiro–Wilks test).

### QTL dissection

The genomic intervals of the mapped QTL were then evaluated in the *Saccharomyces* genome database. The selected candidate genes contained nonsynonymous SNPs (ns-SNPs) in the sequence of the parental strains. The candidate genes were validated by reciprocal hemizygosity analysis according to Steinmetz *et al.* (2002). Briefly, each selected gene was deleted using *Kan-Mx4* cassettes. The deletion cassettes were obtained by PCR amplification of the disruption cassette plus ∼500 pb of the flaking regions using as genomic template the genomic DNA of the strains Y04381, Y06979, Y05455, Y06978, Y04644, Y05451, Y24376 and Y24639, which contain disruption cassettes for the following genes: *PDR1*, *GSC2*, *VMA13*, *MSP1*, *MSB2*, *SRO7*, *PMA1*, and *SEC9*, respectively. The PCR conditions and primers used are listed in File S2. The hybrid strain (HO-BN) was transformed with each deletion cassette using the lithium acetate protocol as described in Gietz and Schiestl (2007). All constructions were verified by insertion PCR. Briefly, the verification consisted of positively amplifying by PCR a fragment containing ∼600 pb of the 5′-flanking region and the 5′-part of the *KanMx4* cassette. All the primers and PCR conditions used for this test are listed in File S2. Once the insertion of the disruption cassette had been verified, we designed a RFLP analysis in order to assess the genotype of the remaining allele. The aim was to obtain a different RFLP profile for each allele of the parental strains. The primers, enzymes, and conditions of each RFLP are listed in File S3. Three distinct clones of both hemizygous hybrids were then tested for their fermentative behavior during the second fermentation, as described before.

### Genetic variability analysis

The allelic frequencies of the four validated genes (*PDR1*, *MSB2VMA13*, and *PMA1*) were estimated among a large set of fully sequenced *S. cerevisiae* strains (97). Amino acid alignments as well as the names of the strains are listed in File S3, File S4, File S5, and File S6. We focused our analysis at the protein level, as ns-SNPs were found for all genes. Putative deleterious effects of ns-SNPs were tested using the SIFT and PROVEAN algorithms (http://sift.jcvi.org) (Table S2) by aligning 144 proteins using the NCBI nonredundant database. The structural alignment of Pma1p was carried out with phyre2 tools (http://www.sbg.bio.ic.ac.uk/phyre2) using default parameters.

### Statistical analysis

All of the statistical and graphical analyses were carried out using R software (R Core Team 2011). The variation of each trait was estimated by the ANOVA using the aov function. Duncan’s multiple comparison test was used to determine which group of means differ significantly (agricolae package) (de Mendiburu 2014). Heterosis was estimated using the following formula:

d/m=(hybrid value−mid parental value)/mid parental value

that measures the phenotypic divergence between the hybrid and the parental strains.

### Data availability

All the strains parents, hybrids and progenies are available under request to the corresponding author. The scripts used as well as the phenotypic and genotypic dataset are given in the supplementary information.

## Results

### Distribution of second fermentation traits reveals heterosis and transgressive segregation

**Figure 1:**
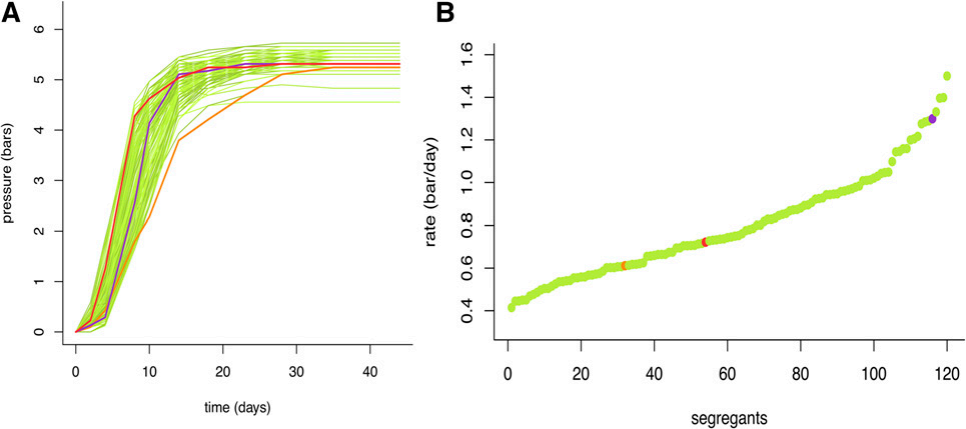
(A) Second fermentation time course for all the strains used. (B) Fermentative rate phenotypic distribution among the segregating population. In orange the parental strain hoGN, in red the parental strain hoSB, in purple their hybrid BN, and in green the 117 segregants.

QTL mapping based on a F1-hybrid design was used to investigate the genetic determinism of the second fermentation kinetics. The 117 progeny clones used were derived from the hybrid BN (hoSB × GN) as previously described (Marullo *et al.* 2007a). The second fermentation kinetics were measured in locked bottles via CO_2_ pressure development over the course of time (Figure 1A). These kinetics were modeled using a 5-parameters logistic fit (5PL model) and five kinetic parameters were extracted. In addition to the maximum pressure (Pmax) and the maximum fermentative “rate,” we investigated the time needed to reach 0.5, 2.0, and 5.0 bars (denoted t0.5, t2, and t5, respectively), representing the initial, middle, and final stage of the fermentation. The phenotypic segregation of the fermentation rate was shown for all the progenies, their relative haploid parental strains (hoGN and hoSB), and the hybrid BN (Figure 1B).

The values of each parameter for the diploid and haploid parental strains are shown in Table 2. The kinetic parameters between haploid and diploid forms did not show significant differences, except for the rate. Haploids exhibited a significantly higher fermentation rate, certainly due to the ploidy level effect on this trait, as reported elsewhere (Salmon 1997; Marullo *et al.* 2006). The ANOVA confirmed that, at the same ploidy level, the parental strains showed significant differences for the rate, t2, and t5. The parental strain SB exhibited faster fermentation, with a higher fermentative rate (0.73 bar/d) and lower t5 (15 d) than the parental GN.

**Table 2:** Phenotypic characterization of the parental strains, their hybrid (HO-BN), and the segregants

	Pmax (bars)	Rate (bar/d)	t0.5 (d)	t2 (d)	t5 (d)
GN	5.33	0.53	4.00	8.60	26.00
SB	5.53	0.73	4.00	7.10	15.00
hoGN	5.50	0.61	5.00	9.00	28.00
hoSB	5.57	0.92	5.00	7.00	13.00
Significance (GN *vs.* SB)		**		**	***
HO-BN	5.37	1.30	3.00	5.00	13.00
d/m	−0.011	1.06	−0.25	−0.36	−0.36
Heritability (%)	90.66	91.72	61.42	90.25	97.85
Transgression (%)	29.91	58.12	32.48	38.46	18.80

Levels of significance are indicated as follows: *** *p* ≤ 0.001, ** *p* ≤ 0.01.

Interestingly, a heterosis effect (*d/m*) between the HO-BN hybrid and the parents (diploids) was observed for all the traits except for Pmax (Table 2). For the rate, the hybrid showed a best parent heterosis effect with a trait value 2.1-fold higher than the mid parental value. This result suggests that both parents contain alleles that can improve fermentation efficiency, thereby providing an opportunity to investigate the molecular bases of heterosis. The continuous distribution observed for all the traits investigated among the segregating clones indicates their polygenic determinism (Figure 1B). Some segregants had phenotypic values outside the parental ranges, with a transgression level varying between 18.8 and 58.12%, depending on the trait (Table 2). This high transgression level reflects that alleles with opposite effects and/or genetic interactions are effective in the BN hybrid. Finally, broad sense trait heritability was > 90% (except for the time needed to reach 0.5), suggesting that the major part of variance captured was genetically determined (Table 2).

### Linkage analysis for second fermentation traits

**Figure 2:**
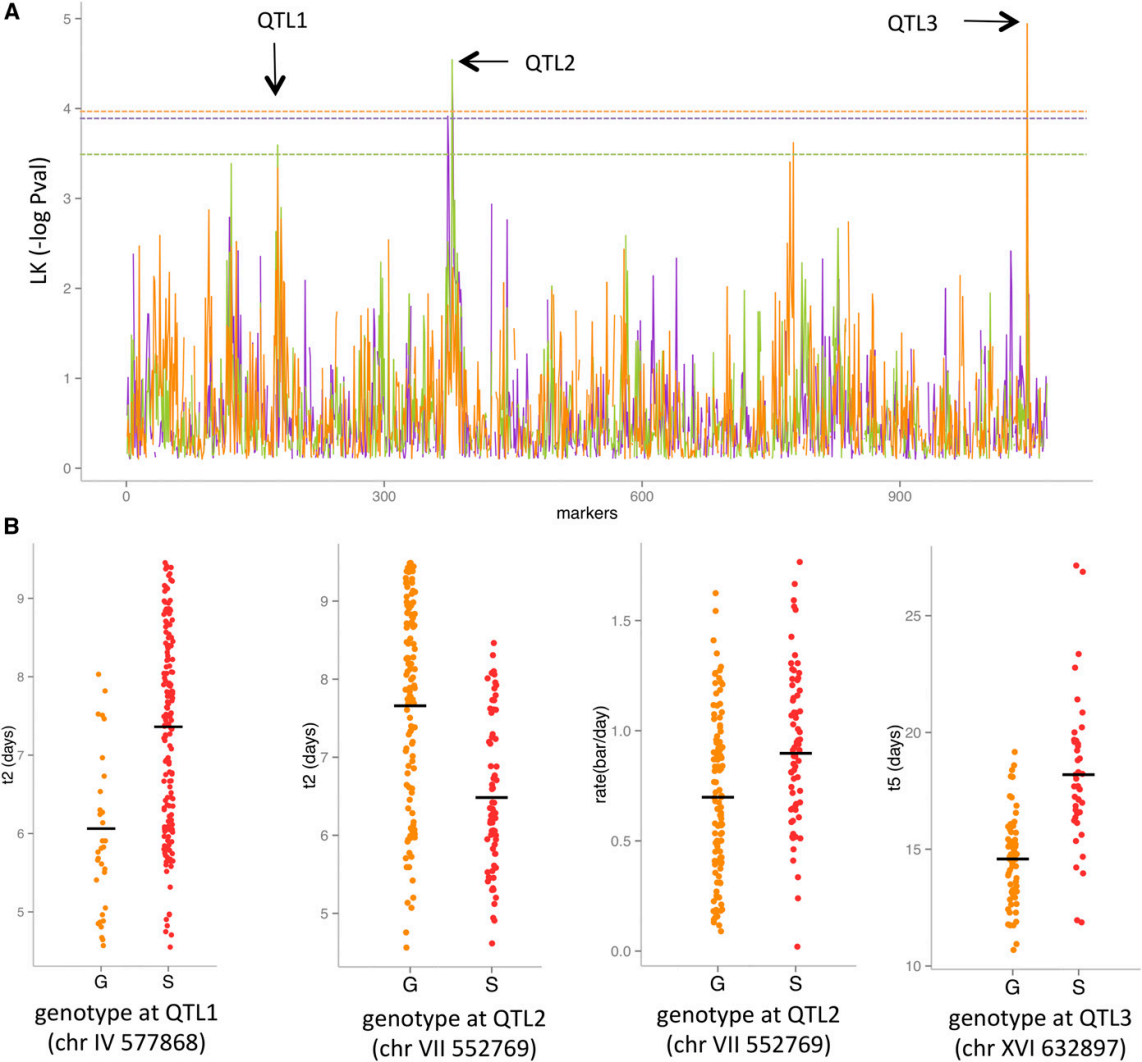
(A) Linkage analysis results for the fermentative rate (purple), t2 (green) and t5 (orange). (B) Segregant phenotypic distributions depending on their genotype at the loci of the mapped QTL. chr, chromosome; LK, linkage; QTL, quantitative trait loci.

The sequencing and genotyping of all the progenies resulted in the construction of a genetic marker map of 1071 markers, evenly distributed, with Mendelian segregation among the progenies. The resulting marker map can be observed in Figure S1. Linkage analysis was conducted by applying the Wilcoxon test. Three QTL localized on distinct chromosomes were detected (FDR < 5%) and linked to three kinetics traits (t2, rate, and t5) (Figure 2A).

The time to reach 2 bars (t2) was linked to both QTL1 (chromosome IV: 564–579 kb, pval = 1.45 × 10^−3^) and QTL2 (chromosome VII: 544–594 bp, pval = 2.88 × 10^−5^). For QTL1, the inheritance of the GN allele conferred a faster fermenting profile while, for QTL2, the inheritance of the SB allele conferred a more rapid CO_2_ release in the initial part of second fermentation. QTL2 was also linked to the fermentation rate, where the SB inheritance promoted a higher fermentation rate. The last QTL (QTL3) was mapped in chromosome XVI (618–654 kb, pval = 1.23 × 10^−05^) in association with the time needed to reach 5 bars (t5), which is related to the final stage of the second fermentation. For this locus, the inheritance of the GN allele had a positive impact on the CO_2_ time course release (Figure 2B).

The variance explained by each QTL and their interactions was estimated by the ANOVA (Table 3). For the phenotypes associated with the initial fermentation stages (t2 and rate), QTL2 had a higher contribution than QTL1. In contrast, QTL3 had a strong effect on the final fermentation stage (t5), explaining ∼50% of the total variance observed. QTL3, although not detected by the linkage test, also explained 14% of t2 total variance. The full statistical model used allowed the detection of significant interactions between QTL2 and the other two QTL. For each trait, the part of variance captured by the ANOVA ranged between 34 and 64%, suggesting that other minor QTL and/or epistatic interactions remain unidentified. The fact that positive alleles were brought by both parental strains could explain the high transgression levels found in BN progeny, as well as the heterosis effect detected.

**Table 3:** Phenotypic variance explained by each QTL detected

Trait	QTL1 (Chr IV)	QTL2 (Chr VII)	QTL3 (Chr XVI)	QTL1 × QTL2	QTL2 × QTL3	QTL1 × QTL3	Cumulated
Rate	**	***		**			34.16%
	2.79%	23.60%		7.76%		
t2	*	***	**				37.73%
	7.80%	15.85%	14.07%			
t5			****		****		63.60%
			49.04%		14.56%	

Levels of significance are indicated as follows: **** *p* ≤ 0.001, *** *p* ≤ 0.01, ** *p* ≤ 0.05, * *p* ≤ 0.1. QTL, quantitative trait locus; Chr, chromosome.

### Dissection of second fermentation QTL

The genomic sequences of mapped QTL were examined in order to identify possible candidate genes. Due to the low contribution of QTL1 (< 8%), we focused the molecular dissection on QTL2 and QTL3. For each single gene found in the QTL regions, the ns-SNPs between SB and GN were tracked. The protein function of candidate genes presenting such polymorphisms was also taken into account. We selected six candidate genes for QTL2: *GSC2*, *MSP1*, *MSB2*, *PDR1*, *PMA1*, and *SEC9*, and two genes, *SRO7* and *VMA13*, for QTL3. The gene functions, as well as the protein sequence changes within the parental strains, are listed in Table 4.

**Table 4:** Candidate genes selected based on their position, function, and the presence of nonsynonymous SNPs in the parental strain sequences

Gene	Function	Changes in the Protein Sequence	QTL
*GSC2*	Catalytic subunit of 1,3-β-glucan synthase, involved in formation of the inner layer of the spore wall	SB: S124P, R1536M, I1502M, L1650F; GN: R382C	QTL2
*MSP1*	Mitochondrial protein involved in sorting of proteins in the mitochondria; putative membrane-spanning ATPase	SB: P38S, T284I	QTL2
*MSB2*	Mucin family member involved in various signaling pathways	SB: S529F	QTL2
*PDR1*	Zinc cluster protein that is a master regulator involved in recruiting other zinc cluster proteins to pleiotropic drug response elements (PDREs) to fine-tune the regulation of multidrug resistance genes	SB: H438Y, F570, N1117K; GN: L955S, K1020N	QTL2
*PMA1*	Plasma membrane H^+^-ATPase, pumps protons out of the cell; major regulator of cytoplasmic pH and plasma membrane potential	SB: H54Q, L176M, D200E, Q283R, V289L, KQ431IE, D718N, E875Q; GN: P74L	QTL2
*SEC9*	t-SNARE protein important for fusion of secretory vesicles with the plasma membrane	SB: QW378XX, NA363DS, EVDHS366(370)SSNXG; deletion: 379-387(WFMDEQQQQ, L465V	QTL2
*SRO7*	Effector of Rab GTPase Sec4p; forms a complex with Sec4p and t-SNARE Sec9p; involved in exocytosis and docking and fusion of post-Golgi vesicles with plasma membrane	SB: I81L, G432A	QTL3
*VMA13*	Subunit H of the V1 peripheral membrane domain of V-ATPase; part of the electrogenic proton pump found throughout the endomembrane system; serves as an activator or a structural stabilizer of the V-ATPase	GN: D120G	QTL3

ATPase, adenosine triphosphatase; GTPase, guanosine triphosphatase; QTL, quantitative trait loci; SNARE, Soluble NSF Attachment Protein REceptor.

**Figure 3:**
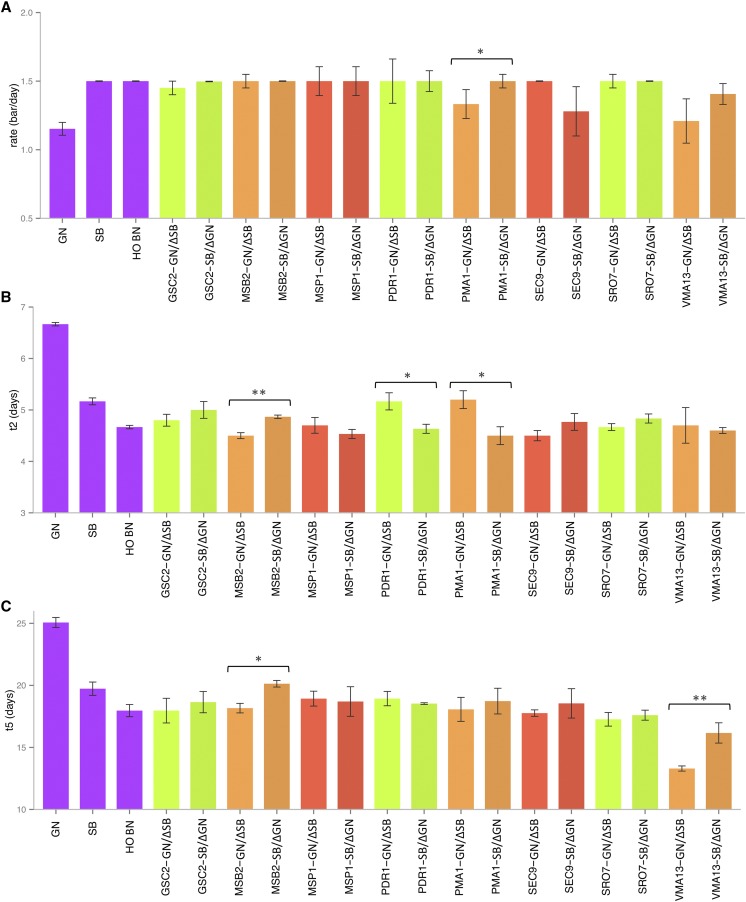
Results of the reciprocal hemizygosity analysis for the fermentative rate (A), t2 (B), and t5 (C). The represented value is the mean of three different biological triplicates; the SE is represented by error bars. An ANOVA was applied to assess the significance of the phenotypic difference between hemizygotes. The level of significance between hemizygotes is indicated as follows: * *p* ≤ 0.05, ** *p* ≤ 0.01, *** *p* ≤ 0.001.

To validate candidate genes, Reciprocal Hemizygosity Analysis (RHA) was performed. Each reciprocal hemizygote, the hybrid strain (HO-BN), and the diploid parental strains (SB and GN), were phenotyped for the second fermentation in three replicates. The rate, t2, and t5 achieved by each hemizygote are represented in Figure 3, A–C, respectively.

**Figure 4:**
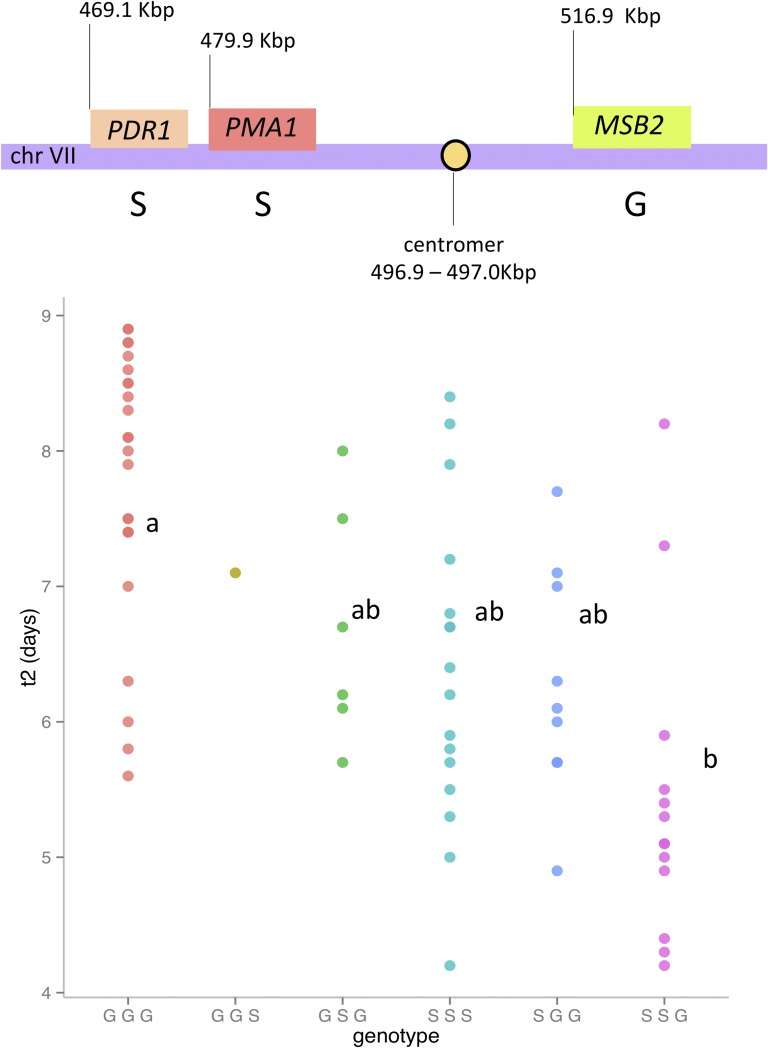
Localization of the genes *PDR1*, *PMA1*, and *MSB2* on chromosome VII; the allele indicated is the favorable one. Phenotypic distribution of the segregants grouped according to their genotype at those three genes. Different letters (a, ab or b) indicate significant differences between groups (significance level, α = 0.05).

The hemizygotes for *PMA1* significantly impacted the fermentation rate. As expected, the hemizygote hybrid carrying the SB allele (*PMA1-SB*/Δ*GN*) had a faster fermentation rate than the one presenting the GN allele (*PMA1-GN*/Δ*SB*). The t2 trait had a more complex genetic determinism. Three genes had an impact on this phenotype (*MSB2*, *PDR1*, and *PMA1*). According to the segregation profile shown in Figure 3B, the hemizygotes containing only the SB allele of *PMA1* and *PDR1* ferment faster than those containing GN alleles, achieving lower t2. Surprisingly, for *MSB2*, the GN allele reduced the value of t2. Consequently, this locus presented a particular feature: three beneficial alleles with alternated inheritance (SB for *PMA1* and *PDR1* and GN for *MSB2*). This particular type of configuration has been previously reported for another locus, generating a heterosis by a pseudooverdominance effect (Steinmetz *et al.* 2002). The ANOVA model of the 117 genotyped progenies indicated that the inheritance of *PMA1*, *PDR1*, and *MSB2* markers explained 4.23, 35.11, and 8.99% of phenotypic variance, respectively. Moreover, a strong interaction effect (40.65%) was found between these genes. Segregant groups with the same parental inheritance for the three genes represent 62.8% of the total genotyped population due to the strong linkage disequilibrium of these loci. However, numerous recombined clones allowed for the effect of each gene to be tested (Figure 4). The inheritance of the specific allelic combination (SSG) for *PDR1*, *PMA1*, and *MSB2* determined the lower average values for t2, in accordance with the RHA. Among the 117 genotyped progenies, only 12 individuals showed this allelic profile. Surprisingly, only one progeny clone has the opposite inheritance profile (GGS). For t5, the RHA indicated that *VMA13* (QTL*3*) and *MSB2* (QTL*2*) genes had a significant effect on this phenotype (Figure 3C). In both cases, the GN inheritance confers faster fermentation kinetics, illustrating the positive contribution of the GN allele set to the second fermentation final stage. The overall combination of positive alleles at different loci constitutes another mechanism of the heterosis observed in the hybrid by a simple dominance effect.

### Exploration of causative mutations

The four genes impacting the second fermentation kinetics (*PDR1*, *MSB2VMA13*, and *PMA1*) showed SNPs and small INDELs (INsertion–DELetions) within the SB and GN parental strains. These punctual genetic variations generate a ns-SNP for each gene (Table S2). For the *MSB2* gene, the nonoptimal parental strain (SB) showed a transition (*C518528T*), resulting in *S529F* amino acid substitution in the extracellular protein part. The *S529F* allele is a rare allele (singleton mutation) that was not found in the GN parent or in any of the other 96 strains analyzed. This substitution could have an effect on protein function according to the Provean analysis (a tool predicting the functional impact of any amino acid substitution based on amino acid conservation). For the *VMA13* gene, we again found a unique transition (*A644194G*) leading to the *D120G* substitution on the strain GN. In this case, this mutation represented the positive allele and was not found in the remaining 97 strains analyzed (including SB).

**Figure 5:**
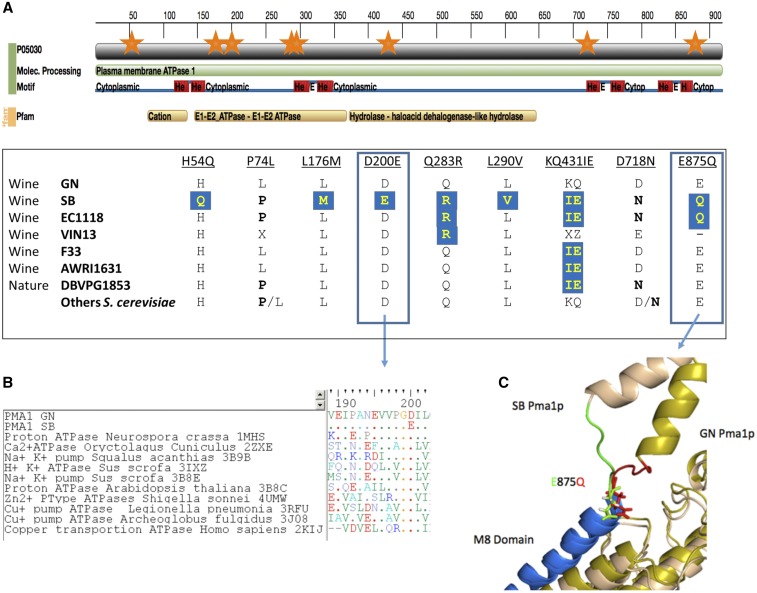
(A) ns-SNPs found within GN and SB on the PMA1 gene. Each orange star represents one ns-SNP; the predicted cytoplasmic and transmembrane regions, as well as the domains, were obtain from the Pfam web site (European Molecular Biology Laboratory-European Bioinformatics Institute). A protein sequence alignment allows the detection of nine ns-SNPs that will be compared to the sequence of 97 *S. cerevisiae* strains (File S6). (B) The *D200E* variation found in strain SB was located in a very conserved region within 144 transmembrane transporters. (C) The *E875Q* allele (SB) impacts the orientation of the fifth transmembrane domain of the protein (3D model carried out with *phyre2* tools). ATPase, adenosine triphosphatase; Cytop., cytoplasmic; Molec., molecular; ns-SNPs, nonsynonymous sequence polymorphisms.

For the essential gene *PMA1*, encoding the membrane ATPase proton pump, drastic genetic variations were found between parental strains. Indeed, nine ns-SNPs were identified between SB and GN (Figure 5A). When compared to the reference genome, eight of these nine substitutions were displayed by the parental strain SB, carrying the positive allele. Interestingly, three of them (H54Q, L176M, and L290V) have not been found in any of the other sequenced strains. Each of the four other substitutions (D200E, Q283R, KQ431IE, and E875Q) is shared by a small subset of strains, including the sparkling wine strain EC1118 (substitution frequencies of 0.02, 0.03, 0.06, and 0.02, respectively). Strikingly, five of the SB alleles provoke a change of residue charge (H54Q, Q283R, KQ431IE, D718N, and E875Q) respective to the GN (and the reference) protein that may affect the transporter proton affinity. Two of them (*D200E* and *E875Q*) were predicted to affect the protein function by Provean and/or SITF algorithm (File S1). The *D200E* substitution occurs in a very conserved region of the E1–E2 ATPase domain (Pfam 00122). Although aspartate and glutamine residues are functionally similar, only three proteins showing this substitution are found in 552 proteins belonging to a long range of organisms (*psy* blast alignment on uniprot database) (Figure 5B). The *E875Q* substitution occurs in the membrane segment M8, and was predicted to modify the Pma1p structure of the SB protein using *phyre2* tools (Figure 5C).

The impact of *PDR1* polymorphisms is more difficult to decipher. Pdr1p parental sequences showed an INDEL of five asparagines occurring in a N-rich region highly variable within the 97 strains compared. In addition, three single amino acid polymorphisms were found. The substitution *L955S* is common to GN and two other strains, while *H438Y* and *F571L* are unique to SB, the positive parental strain. These last two polymorphisms are located in a fungal transcription factor domain (Pfam 04082) and the substitution *F571L* was predicted to affect the Pdr1p transcriptional activity.

### GxE interactions, the effect of the base wine pH

**Figure 6:**
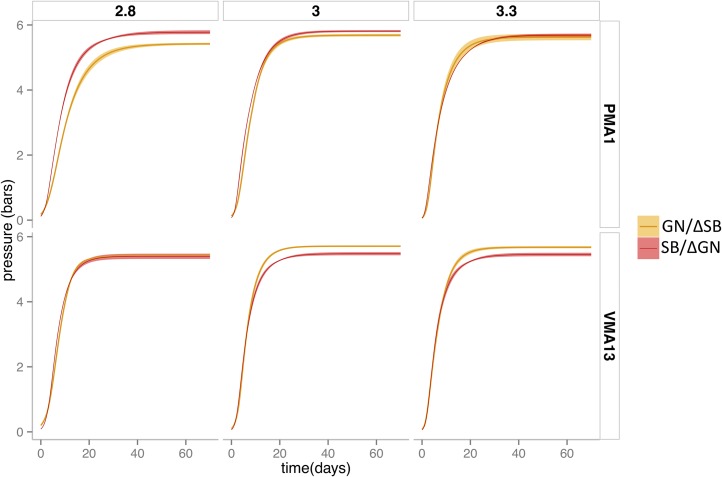
Kinetic curves of the hemizygotes for *PMA1* and *VMA13*, and the diploid hybrid strain BN obtained when fermenting base wine at different pH: 2.8, 3.0, and 3.3. The kinetic curve is the mean between triplicates and the shadow around the line represents the SE.

The roles of two genes encoding for ATPase protein complexes involved in pH homeostasis (*PMA1* and *VMA13*) were validated. Given the increased ethanol toxicity at low pH values (Alexandre and Charpentier 1998), commonly featured in base wines, this environmental factor represents one of the major constrains for successful second fermentation (completion/progress). Consequently, minimal variations of pH in base wine may have important consequences on yeast physiology. The pH of base wine was modified (2.8, 3.0, and 3.3) and the phenotypic response of hemizygous hybrids for *PMA1* and *VMA13* genes was tested for all the kinetic parameters investigated in this study (Table 5). For *PMA1* gene, the beneficial effect of the SB allele increased at low pH (Figure 6). At pH = 2.8, this gene affected all the kinetic parameters, while at pH = 3.3 the positive impact of this allele was detected only for the first fermentation part (rate and t0.5). The gene *VMA13* exhibited a more complex interaction with the base wine pH (Figure 6). At pH = 2.8, the SB allele was the most beneficial and had a significantly positive contribution on the first part of the fermentation (rate, t0.5, and t2) (Table 5). In contrast, for the last part of the second fermentation (t5 and Pmax) the GN allele has a positive effect. Altogether, pH variations indicate that both SB and GN alleles have beneficial effects for different kinetic parameters. At lower pH, the SB alleles of *PMA1* and *VMA13* promoted a faster fermentation start. At higher pH, the GN allele of *VMA13* also had a positive effect, accelerating the last part of the fermentation. Assuming the fact both *PMA1^SB^* and *VMA13^GN^* are mostly dominant (Figure 3), this switching effect might result in a higher robustness of heterozygous individuals to pH variations.

**Table 5:** Values of each kinetic parameter, plus their standard error, in every pH condition studied for both hemizygotes (*PMA1* and *VMA13*)

Gene	pH	Trait	GN/ΔSB	ΔGN/SB	Significance
*PMA1*	2.8	Pmax	5.49 ± 0.03	5.82 ± 0.06	**
		Rate	0.54 ± 0.04	0.91 ± 0.13	*
		t0.5	2.56 ± 0.08	2.23 ± 0.03	*
		t2	7.46 ± 0.07	5.6 ± 0.06	***
		t5	27.13 ± 2.14	17.5 ± 0.72	*
	3	Pmax	5.74 ± 0.04	5.88 ± 0.03	
		Rate	0.69 ± 0.07	1.27 ± 0.18	*
		t0.5	2.46 ± 0.03	2 ± 0.08	*
		t2	5.8 ± 0.15	4.6 ± 0.16	*
		t5	15.53 ± 0.63	14.6 ± 0.41	
	3.3	Pmax	5.64 ± 0.09	5.73 ± 0.04	
		Rate	1.15 ± 0.06	1.5 ± 0.01	**
		t0.5	2.33 ± 0.06	1.86 ± 0.03	**
		t2	5.1 ± 0.2	4.6 ± 0.01	
		t5	17.03 ± 1.98	14.6 ± 0.05	
*VMA13*	2.8	Pmax	5.49 ± 0.02	5.42 ± 0.08	
		Rate	0.51 ± 0.05	0.84 ± 0.03	**
		t0.5	2.66 ± 0.03	2.53 ± 0.03	**
		t2	6.1 ± 0.15	5.3 ± 0.03	**
		t5	14.9 ± 0.40	16.1 ± 0.82	
	3	Pmax	5.78 ± 0.02	5.53 ± 0.05	**
		Rate	0.92 ± 0.10	1.13 ± 0.05	
		t0.5	2.36 ± 0.07	2.23 ± 0.08	
		t2	5 ± 0.15	4.76 ± 0.14	
		t5	12.7 ± 0.46	15.4 ± 0.50	**
	3.3	Pmax	5.74 ± 0.03	5.50 ± 0.05	*
		Rate	1.15 ± 0.17	1.36 ± 0.06	
		t0.5	2.06 ± 0.09	2 ± 0.05	
		t2	4.57 ± 0.14	4.43 ± 0.14	
		t5	13.46 ± 0.67	15.73 ± 0.67	*

Significance is indicated as follows: *** *p* ≤ 0.001, ** *p* ≤ 0.01, * *p* ≤ 0.05.

## Discussion

### Identification of natural genetic variations involved in pH homeostasis and the stress response

Deciphering the genetic mechanisms controlling natural trait variation is one of the major frontiers in genetics. *S. cerevisiae* is a promising organism for bridging the phenotype–genotype gap (Liti and Louis 2012). In fundamental science, quantitative genetics allows such genetic paradigms as missing heritability (Bloom 2013) or epigenetic-control inheritance (Nagarajan 2010, Filleton *et al.* 2015) to come to the fore. In a more applied field, QTL mapping is very useful to understand the natural genetic variations modulating the performance of industrial strains in specific contexts. For enological traits, this strategy has been prolific in several aspects, including undesirable compound production (Salinas *et al.* 2012; Gutiérrez *et al.* 2013; Jara *et al.* 2014). Moreover, QTL mapping identifies natural genetic variations that can subsequently be used in molecular breeding approaches (Marullo *et al.* 2007b; Dufour *et al.* 2013). Identifying those variations can be useful in improving our current understanding of cellular and biochemical mechanisms. In the current study, the genetic basis of oenological traits pertaining to the second fermentation was analyzed. The parental strains used were derived from industrial wine starters and showed strong differences in oenological traits (Marullo *et al.* 2006, 2007a; Zimmer *et al.* 2014), including their kinetics during the second fermentation (Martí-Raga *et al.* 2015). We identified four genes (*PMA1*, *PDR1*, *MSB2*, and *VMA13*), whose allelic variation affects second fermentation kinetics. Their functions are mainly related to cellular homeostasis. First, *PMA1* is an essential gene that encodes the principal membrane ATPase, the main agent regulating the intracellular pH. Different studies with Pma1p mutants have shown that punctual mutations result in an inability of strains to grow at low pH or in the presence of weak acids, suggesting a reduced ability to extrude protons from the cell [recently reviewed by Orij *et al.* (2011)]. The allelic variation within *PMA1* may cause a reduction its enzymatic activity. Considering the low pH found in base wine, this weak enzymatic activity may affect both the intracellular pH and the fermentative efficiency of yeast. The sequence analysis of this proton transporter reveals nine amino acid substitutions within the parental strains. Two of them (*D200E* and *E875Q*) are predicted to affect the Pma1p function and are specifically found within another industrial genetic background (EC1118) belonging to the Champagne cluster (Novo *et al.* 2009). Interestingly, we validated a second proton pump gene, *VMA13*, that encodes for the V1-subunit of the vacuolar ATPase (*V-ATPase*), harboring the sites for ATP hydrolysis. The V-ATPase is involved in the control of both vacuolar and cytosolic pH by pumping protons from the vacuole to the cytosol (Orij *et al.* 2011), and its activity has an impact on Pma1p expression and localization (Martínez-Muñoz and Kane 2008). In an oenological context, *VMA13* has been identified as one of the 93 essential genes needed to maintain the complete fermentation (Walker *et al.* 2014). Furthermore, this protein has been associated with ethanol resistance, thus, strains lacking *VMA13* are more sensitive to ethanol (Fujita *et al.* 2006; Teixeira *et al.* 2009). In this study, *VMA13* showed one unique, rare allelic variation (*D120G*) that could explain the phenotypic discrepancy observed within the parental strains. The V-ATPase works in parallel with the HOG (High Osmolarity Glycerol) pathway in order to adapt yeast cells to osmotic stress (Li *et al.* 2012). The third gene identified, *MSB2*, is a mucine family member acting as an osmosensor in the *Sho1p-mediated* HOG pathway. Therefore, this transmembrane sensor plays a central role in adapting yeast cells to osmolarity changes (Hohmann 2009). Furthermore, the HOG pathway plays a role in yeast adaptation to other stress sources, such as cold and acidic environments, that are particularly prevalent in second fermentation (Hayashi and Maeda 2006; Mollapour and Piper 2007).

The last gene impacting the second fermentation kinetics is *PDR1*. This gene encodes a transcription factor controlling the expression of several plasma membrane transporters that belong to the superfamily of ATP-binding cassettes (ABC), including Pdr5p, Yor1p, and Pdr10p. These pleiotropic drug-response proteins (Balzi *et al.* 1987) detoxify the yeast cell and transport several compounds, including phospholipids, peptides, and sterols. Hence, they are thought to be implicated in controlling membrane lipid homeostasis, the regulation of membrane permeability, and the phospholipid bilayer distribution (Wilcox *et al.* 2002; Li and Prinz 2004; Jungwirth and Kuchler 2006). The maintenance of membrane composition and fluidity is of special relevance during the alcoholic fermentation, due to its impact on yeast fermentative performance and viability (Torija *et al.* 2003; Beltran *et al.* 2008).

The fermentation performances of sake yeast are modulated by the expression level of *PDR1* and its paralog *PDR3*, and/or by mutations affecting the ABC transporters they regulate (Mizoguchi *et al.* 2002; Watanabe *et al.* 2000). Consequently, changes in the *PDR1* sequences could result in higher/lower transcription factor activity affecting cell homeostasis, thereby modulating the viability and fermentative capacity of yeast.

We identified four genes that play a key role in various stress responses, including pH and lipid homeostasis, ethanol and low temperature resistance, and osmotic pressure. These stressful conditions are found during the second fermentation, and may have promoted the emergence of adapted alleles for these specific pathways. Except for *MSB2*, all the genes showing positive allele(s) are found with a low frequency in the yeast population (< 6%). This suggests that many other possible adaptive mutations may have been selected, but these still need to be identified.

### Resolution at the molecular level of a novel case of heterosis

Recent studies on yeast aimed at deciphering the hybridization effect on a vast range of phenotypic traits conclude that inter- and intraspecific hybridization can result in heterosis (Plech, de Visser, and Korona 2013; da Silva *et al.* 2015; Zörgö *et al.* 2012; Shapira *et al.* 2014). In this genetic mapping study, we detected and characterized the heterosis effect observed for fermentation traits at the gene level. Quantitative genetics studies show that heterosis may be attributable to dominance (Xiao *et al.* 1995; Cockerham and Zeng 1996), overdominance (Stuber *et al.* 1992; Li *et al.* 2001; Luo *et al*., 2001), pseudooverdominance (Crow 1998; Lippman and Zamir 2007), and/or epistasis (Schnell and Cockerham 1992; Li *et al.* 2001). The present study sheds light on new examples of dominance and pseudooverdominance. For the three mapped QTL, the positive alleles were apparently given by strain GN (QTL1 and QTL3) and by strain SB (QTL2) (Figure 2B). QTL1 and QTL2 were related to the first part of the fermentation, while QTL3 impacted the total time needed to finish fermentation. The combination of one positive copy of each QTL in the hybrid explains the hybrid vigor observed. As the three QTL are not genetically linked, a large portion of the F1 haploid population presents transgressive values with respect to the parental strains. In addition to this basic dominance effect, a particular pseudooverdominance effect was found for the QTL2 identified. This locus impacts the traits rate and t2, which represent the middle fermentation. Reciprocal hemizygosity analysis showed that the genetic determinism of t2 was positively enhanced by three alleles *PMA1^SB^*, *PDR1^SB^*, and *MSB2^GN^*. The heterosis observed in the hybrid strain constitutes a second example of pseudooverdominance heterosis described in *S. cerevisiae*. Those particular loci can be revealed when the distinct populations (that have evolved separately over a long period) interbreed (Liti and Louis 2012). The strong physical linkage of these three genes, and the fact that positive alleles are quite rare, strongly limits the chance of finding this optimal allele set in a natural isolate. As the parental strains GN and SB are derived from wine isolates that are genetically divergent (Richards *et al.* 2009), their hybridization promoted the association of positive alleles that have been generated by different selection events. This situation may explain why heterosis is prevalent among domesticated populations (Plech *et al.* 2013). The SNP markers of *PMA1*, *PDR1*, and *MSB2* showed Mendelian segregation for the 117 genotyped progenies (χ^2^ test > 0.05); however, an unexpected misbalanced inheritance for two specific haplotypes was found. Indeed, pattern SSG, which conferred the best phenotype, was found 12 times more than reciprocal pattern GGS. Although the spore clones used in this study were obtained by tetrad microdissection in a nonselective medium (YPD), this GGS pattern might be counterselected as a result of genetic interactions, as previously reported (Hou *et al.* 2015). The presence of *CEN7* between *PMA1* and *MSB2* might influence this phenomenon by chromosome interference and/or gene conversion.

### Allele × pH interactions suggest that heterozygosity promotes a strong phenotypic robustness

As two of the validated genes, *PMA1* and *VMA13*, are ATPases involved in intracellular pH homeostasis, the effect of the pH of base wine was investigated. Although relatively small (± 0.5 pH units), the range of pH explored has an important physiological impact in the oenological context, affecting the completion of second fermentation. Depending on the pH, the allelic variations of *PMA1* and *VMA13* modulated the fermentation kinetics in a complex way. For *PMA1*, the SB allele had a positive effect in low pH conditions whereas, for *VMA13*, the positive allele changed in relation to the pH. The SB allele conferred better fitness at pH = 2.8, but in mild conditions (intermediate and higher pH) the GN allele was the beneficial one. The combination of both genotypes *PMA1^SB^* and *VMA13^GN^* in the hybrid BN could result in a better adaptation to a larger range of pH (between 2.5 and 4.5 in grape juice), suggesting that hybridization may confer phenotypic robustness. This observation is in accordance with a recent study showing that intra- and interspecific hybridization generate a global phenotypic homeostasis in a winemaking context (da Silva *et al.* 2015).

### Conclusions

In the present study, we applied a QTL mapping approach to decipher the genetic determinism of a complex industrial trait of economic interest. Thanks to the high quality of the genetic map generated, we found the impact of four genes to have a key function in cellular homeostasis. Interestingly, the allele combination of favorable alleles generates a strong heterosis effect in the hybrid due to dominance and pseudooverdominance effects. Finally, we observed that the heterozygous status of the hybrid for *PMA1* and *VMA13* provides more phenotypic robustness due to genetic × environment interactions between PMA1 and VMA13 genes with the pH of base wine. All these data illustrate the complexity of genetic determinism of quantitative traits and pave the way to improve yeast strains for fermentation applications.

## Supplementary Material

Supplemental material is available online at www.g3journal.org/lookup/suppl/doi:10.1534/g3.116.037283/-/DC1.
