# Supplementary figures and images for: Genetic Causes of Phenotypic Adaptation to the Second Fermentation of Sparkling Wines in Saccharomyces cerevisiae
Source: G3 (Bethesda). 2016 Nov 28;7(2):399–412. doi: 10.1534/g3.116.037283 (PMC5295589; doi:10.1534/g3.116.037283)

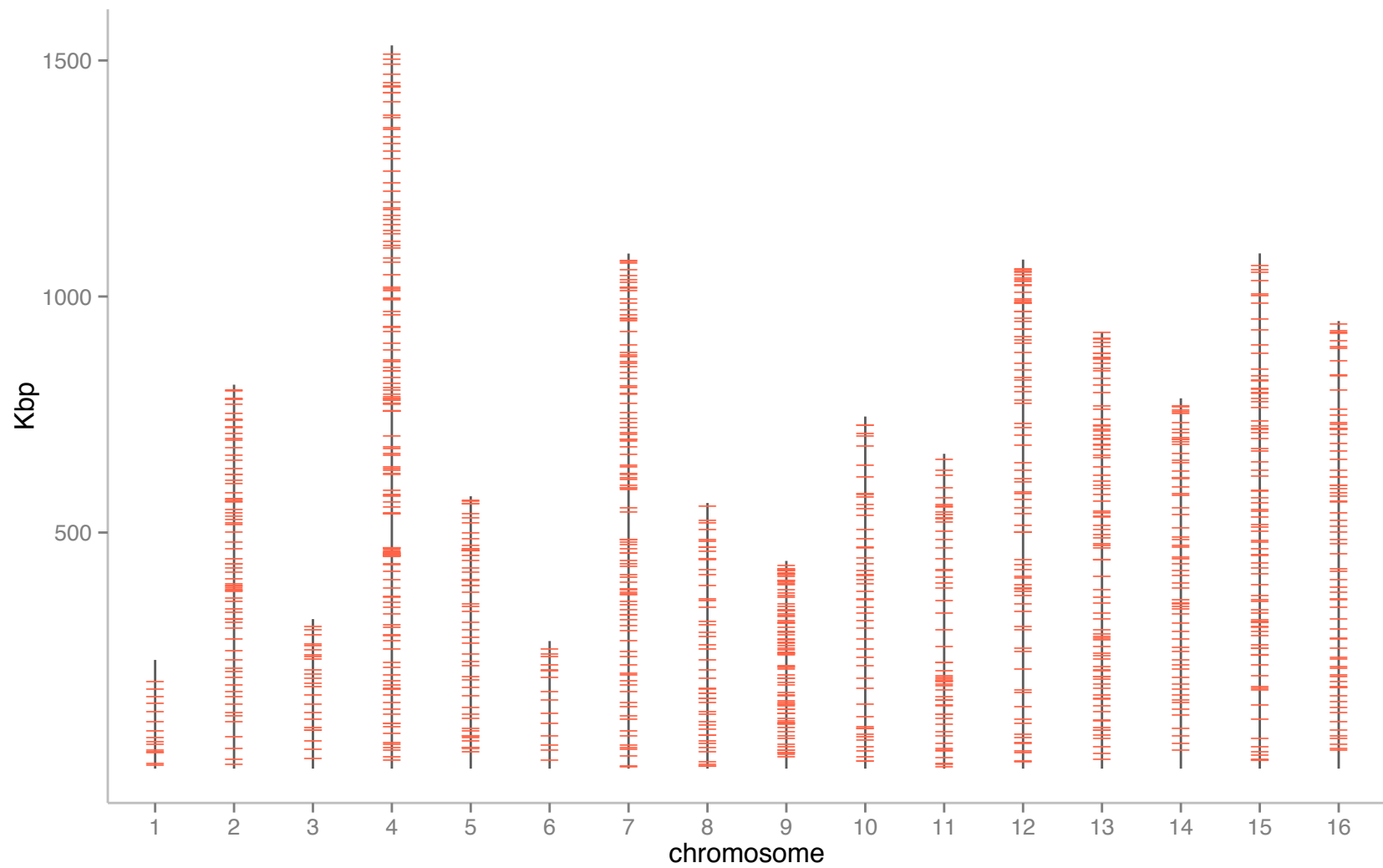

Supplement: Supplementary file 1 [file 399FigureS1.pdf]
