# Supplementary material for: Genetic Causes of Phenotypic Adaptation to the Second Fermentation of Sparkling Wines in Saccharomyces cerevisiae
Source: G3 (Bethesda). 2016 Nov 28;7(2):399–412. doi: 10.1534/g3.116.037283 (PMC5295589; doi:10.1534/g3.116.037283)
Supplement: Supplementary file 2 [file 399FileS1.docx]

# File S1 R SCRIPTS

## 1. SCRIPT USED TO OBTAIN NON-COMMON SNPs FROM PARENTAL STRAINS

#extraction of SNP from data sequence. Starting from the SnpEffect file obtained for each parenatl strain

cheminacces<-"/Users/maria/Dropbox/Second FAL/QTL/"

read.delim(paste(cheminacces, "microarray/sace-SC_SBsnpEff.txt",sep=""))->SBtab

read.delim(paste(cheminacces, "microarray/sace-SC_GNsnpEff.txt",sep=""))->GNtab

#compile les SNP homozygotes with differents effects "SYNONYMOUS_CODING", NON_SYNONYMOUS_CODING, STOP_LOST, CODON_INSERTION, CODON_DELETION, START_LOST, STOP_GAINED

which(SBtab$Effect=="SYNONYMOUS_CODING"|SBtab$Effect=="NON_SYNONYMOUS_CODING"|SBtab$Effect=="STOP_LOST"|SBtab$Effect=="CODON_INSERTION"|SBtab$Effect=="CODON_DELETION"|SBtab$Effect=="START_LOST"|SBtab$Effect=="STOP_GAINED")->SBSAPlist

which(GNtab$Effect=="SYNONYMOUS_CODING"|GNtab$Effect=="NON_SYNONYMOUS_CODING"|GNtab$Effect=="STOP_LOST"|GNtab$Effect=="CODON_INSERTION"|GNtab$Effect=="CODON_DELETION"|GNtab$Effect=="START_LOST"|GNtab$Effect=="STOP_GAINED")->GNSAPlist

SBtab[SBSAPlist,]->SAPSB

GNtab[GNSAPlist,]->SAPGN

#new strategy: select SNPs directly #find SNP against BY that are common to GN and SB

#Creation of two tables: one for GN and another on for SB. In each table: chromosome, position, reference and change, homozigous, quality and two supplementary columns for later use.

cbind(as.numeric(SAPGN[,"X..Chromo"]),as.numeric(SAPGN[,"Position"]),as.character(SAPGN[,"Reference"]),as.character(SAPGN[,"Change"]),as.character(SAPGN[,"Homozygous"]),as.character(SAPGN[,"Quality"]),as.character(SAPGN[,"Gene_name"]),as.character(SAPGN[,"Gene_name"]))->PG

colnames(PG)<-c("chr","pos","ref","change", "homozigous","quality","gene","gene")

cbind(as.numeric(SAPSB[,"X..Chromo"]),as.numeric(SAPSB[,"Position"]),as.character(SAPSB[,"Reference"]),as.character(SAPSB[,"Change"]),as.character(SAPSB[,"Homozygous"]),as.character(SAPSB[,"Quality"]),as.character(SAPSB[,"Gene_name"]),as.character(SAPSB[,"Gene_name"]))->PS

colnames(PS)<-c("chr","pos","ref","change", "homozigous","quality","gene","gene")

#create a word for each position and change and chromosome.

for (i in 1:dim(PG)[1]) {

paste(PG[i,"chr"],"_",PG[i,"pos"],"_",PG[i,"ref"],"_",PG[i,"change"])->PG[i,8]

PG[i,7]<-"GN"

}

for (i in 1:dim(PS)[1]) {

paste(PS[i,"chr"],"_",PS[i,"pos"],"_",PS[i,"ref"],"_",PS[i,"change"])->PS[i,8]

PS[i,7]<-"SB"

}

colnames(PG)[7:8]<-c("strain","word")

colnames(PS)[7:8]<-c("strain","word")

#write only unique entries

PG[c(which(!duplicated(PG[,"word"]))),]->PG

PS[c(which(!duplicated(PS[,"word"]))),]->PS

#delete entries type of: * #NOM?

which(PS[,"ref"]=="*")->a

PS[-a,]->PS

which(PG[,"ref"]=="*")->a

PG[-a,]->PG

#write and read the tables to avoid problems later on

write.table(PS, file=paste(cheminacces,"PS.csv", sep=""),sep=";", na="NA", dec=".", row.names=F, col.names=T)

write.table(PG, file=paste(cheminacces,"PG.csv", sep=""),sep=";", na="NA", dec=".", row.names=F, col.names=T)

read.csv(paste(cheminacces,"PS.csv", sep=""), header=T, sep=";", dec=".",na.strings="NA")->PS

read.csv(paste(cheminacces,"PG.csv", sep=""), header=T, sep=";", dec=".",na.strings="NA")->PG

#Now we are looking for unique entries (or not duplicated). The unique entries will be the SNP that are not common to both GN and SB, so the SNP existing in between both of them.

which(PG[,"word"]%in%PS[,"word"])->commonGN

PG[-commonGN,]->SNPGN

#GN SNP

dim(SNPGN)[1]

#SNP GN: 6756

which(PS[,"word"]%in%PG[,"word"])->commonSB

PS[-commonSB,]->SNPSB

#SB SNP

dim(SNPSB)[1]

#SNP SB: 6361

#Total number of SNPs

dim(SNPSB)[1]+dim(SNPGN)[1]

#13117

#eliminate duplicated entries refering chromosome and position.

paste(SNPGN[,"chr"], SNPGN[,"pos"], sep="_")->SNPGN[,"word"]

paste(SNPSB[,"chr"], SNPSB[,"pos"], sep="_")->SNPSB[,"word"]

write.table(SNPSB, file=paste(cheminacces,"PS_SBsnp.csv", sep=""),sep=";", na="NA", dec=".", row.names=F, col.names=T)

write.table(SNPGN, file=paste(cheminacces,"PG_GNsnp.csv", sep=""),sep=";", na="NA", dec=".", row.names=F, col.names=T)

## 2. SCRIPT USED TO GENOTYPE PROGENIES (supplementary material S3)

#Read file containing all SNP of interest. (Not common between parental strains)

path<-"/Users/maria/Desktop/marker selection/"

#file parentals

read.csv(paste(path, "SNP_GNSB.csv",sep=""),sep=";")->GNSB

GNSB[,c(1,2,3,4,7,8)]->GNSB

########################################################################################################

#Read file for each progenie. (Obtained pileup data set, contains all mapped positions)

c("23","24","39","42","56","59","77","84","92","93","94","108","144","145","149","170","177","193","194","195","196","197","198","199","200","201","202","203","204","205","206","207","208","209","210","211","212","213","214","215","216","217","218","219","220","221","222","223","224","225","226","227","228","229","230","231","232","233","234","235","236","237","238","239","240","241","242","243","244","245","246","247","248","249","250","251","252","253","254","255")->Open

for (j in 1:length(Open)){

read.delim(paste(path,paste(Open[j],"all.txt",sep=""),sep=""),header=F,sep="\t")->pro

colnames(pro)<-c("chr","pos","ref","change")

gsub(">", "", pro[,"chr"])->pro$chr

#change [chromsome=1] for 1

which(pro$chr=="[chromosome=1]")->c1

pro[c1,"chr"]<-1

which(pro$chr=="[chromosome=2]")->c1

pro[c1,"chr"]<-2

which(pro$chr=="[chromosome=3]")->c1

pro[c1,"chr"]<-3

which(pro$chr=="[chromosome=4]")->c1

pro[c1,"chr"]<-4

which(pro$chr=="[chromosome=5]")->c1

pro[c1,"chr"]<-5

which(pro$chr=="[chromosome=6]")->c1

pro[c1,"chr"]<-6

which(pro$chr=="[chromosome=7]")->c1

pro[c1,"chr"]<-7

which(pro$chr=="[chromosome=8]")->c1

pro[c1,"chr"]<-8

which(pro$chr=="[chromosome=9]")->c1

pro[c1,"chr"]<-9

which(pro$chr=="[chromosome=10]")->c1

pro[c1,"chr"]<-10

which(pro$chr=="[chromosome=11]")->c1

pro[c1,"chr"]<-11

which(pro$chr=="[chromosome=12]")->c1

pro[c1,"chr"]<-12

which(pro$chr=="[chromosome=13]")->c1

pro[c1,"chr"]<-13

which(pro$chr=="[chromosome=14]")->c1

pro[c1,"chr"]<-14

which(pro$chr=="[chromosome=15]")->c1

pro[c1,"chr"]<-15

which(pro$chr=="[chromosome=16]")->c1

pro[c1,"chr"]<-16

#contsruct big word to compare sets:

paste(pro[,"chr"],pro[,"pos"], sep="_")->word.pro

which(word.pro%in%GNSB[,"word"])->row.pro

pro[row.pro,]->pro

#write progenie file.

write.table(pro, file=paste(path,Open[j],"snp.csv", sep=""),sep=";", na="NA", dec=".", row.names=F, col.names=F)

rm(list=c("c1","pro","word.pro", "row.pro"))

}

rm(list=ls())

#Second loop to actually genotype the progenie.

path<-"/Users/maria/Desktop/marker selection/"

read.csv(paste(path, "SNP_GNSB.csv",sep=""),sep=";")->GNSB

GNSB[,c(1,2,3,4,7,8)]->GNSB

c("23","24","39","42","56","59","77","84","92","93","94","108","144","145","149","170","177","193","194","195","196","197","198","199","200","201","202","203","204","205","206","207","208","209","210","211","212","213","214","215","216","217","218","219","220","221","222","223","224","225","226","227","228","229","230","231","232","233","234","235","236","237","238","239","240","241","242","243","244","245","246","247","248","249","250","251","252","253","254","255")->Open

matrix(data=NA, nrow=dim(GNSB)[1], ncol=length(Open))->Mar

cbind(GNSB,Mar)->mar

rm(list=c("GNSB", "Mar"))

for(j in 1:length(Open)){

read.csv(paste(path,Open[j],"snp.csv",sep=""),sep=";")->pro

colnames(pro)<-c("chr","pos","ref","change")

paste(pro$chr, pro$pos, sep="_")->word

cbind(pro,word)->pro

colnames(pro)[5]<-"word"

for(i in 1:dim(mar)[1]){

which(pro$word%in%mar$word[i])->a

if(length(a)!=0){

if(pro[a,"change"]!="N"){

if(as.character(pro[a,"ref"])==as.character(mar[i,"ref"])){

if(as.character(pro[a,"change"])==as.character(mar[i,"change"])){

if(mar[i,"strain"]=="GN") {"G"->mar[i,6+j]}

if(mar[i,"strain"]=="SB") {"S"->mar[i,6+j]}

}

if(as.character(pro[a,"change"])!=as.character(mar[i,"change"])){

if(as.character(pro[a,"change"])==as.character(pro[a,"ref"])){

if(mar[i,"strain"]=="GN") {"S"->mar[i,6+j]}

if(mar[i,"strain"]=="SB") {"G"->mar[i,6+j]}

}}}}}

colnames(mar)[6+j]<-Open[j]

}

}

write.table(mar, file=paste(path,"markers_hap.csv", sep=""),sep=";", na="NA", dec=".", row.names=F, col.names=F)

## 3. SCRIPT USED TO PERFORM QTL MAPPING (supplementytary material S4)

#### Script to perform QTL detection not assuming the data is distributed in a normal way.

#Steps to follow:

# For each phenotyping trait (columns 2:XXX):

#a) Apply wilcoxon test on phenotipic trait depending on genotype (all markers) x~y.

#b) Keep the pvalue.

#c) 1000 permutations:

#c.1)randomize the phenotype sample(y).

#c.2)apply wilcoxon test on randomized phenotipic trait genotype x~y for all markers. Keep the lowest value.

#d)At the end: list with the lowest tests statistics obatined for each permutation for every marker.

#e)Order them from lowest to highest. Select 50th value (5%)->Significant thershold.

#f)Which marker has a lowest test statistic? QTL!

#g)Plot and keep results

source("http://bioconductor.org/biocLite.R")

biocLite("ggbio")

# Define directory where the file containning the phenotype and genotype is located

path<-"/Users/maria/Dropbox/Second FAL/QTL/QTL detection 1000 markers/"

#Read file:

read.csv(paste(path, "tQTL_NGS.csv", sep=""), header=FALSE, sep=";", dec=".")->QTL

library(ggplot2)

library(GenomicRanges)

library("BiocInstaller")

biocLite("ggbio")

library(ggbio)

#Start loop to analyse each phenotype:XXXXXX (phenotype columns). BE CAUTIONS WITH THE LENGHT OF VECTOR REAL. ADJUST to the number of markers contained in the file

for( i in 2:XXXX){

vector(mode="logical", length=dim(QTL)[2]-XXX)->real

vector(mode="logical", length=1000)->thershold

if(length(which(is.na(QTL[,i])))!=0){

QTL[-which(is.na(QTL[,i])),]->qtl

}

if(length(which(is.na(QTL[,i])))==0){

QTL->qtl

}

#loop.For each marker.(markers colons) p is marker1. Real p.val test phenotype~genotype.

real[1:length(real)]<-1

#BE CAUTIONS WITH THE P STARTING THE LOOP. MAY NEED TO BE ADJUSTED

p<-48

for (p in 48:dim(qtl)[2]){

#apply wilcox test on the pehnotypic data

if(length(which(qtl[-c(1:2),p]=="S"))!=0 && length(which(qtl[-c(1:2),p]=="G"))!=0 ){

wilcox.test(as.numeric(as.character(qtl[-c(1:2),i]))~as.vector(qtl[-c(1:2),p]))$p.val->real[p-47]

}

}

# Start permutations

for(j in 1:1000){

vector(mode="logical", length=dim(qtl)[2]-47)->permutation

permutation[1:length(permutation)]<-1

#randomize phenotype

sample(qtl[-c(1:2),i])->random

#Calculate for each marker the wilcoxon test. Write it in the vector of permutations

for (p in 48:dim(qtl)[2]){

if(length(which(qtl[-c(1:2),p]=="S"))!=0 && length(which(qtl[-c(1:2),p]=="G"))!=0 ){

wilcox.test(as.numeric(as.character(random))~as.vector(qtl[-c(1:2),p]))$p.val->permutation[p-47]

}

}

#Select the lowest value for all markers obtained in each permutation. Write it in the vector with lowest p.val (thershold)

min(permutation, na.rm=T)->thershold[j]

print(j)

}

#After permutations: order thershold from lowest to highest.

thershold[order(thershold)]->thershold

thershold[50]->def.thershold1

-log10(def.thershold1)->def.thershold

#plot-it!. Be cautions. Plotting against marker, May need adjustment, the colons deleted correspond to the phenotype data.

chr<-as.numeric(as.character(t(QTL[1, -c(1:47)])))

start<-as.numeric(as.character(t(QTL[2,-c(1:47)])))

end<-as.numeric(as.character(t(QTL[2,-c(1:47)])))

strand<-rep("*", length(start))

paste("M", seq(1:length(start)))->marker

-log10(real)->real1

gr<-GRanges(seqnames=Rle(chr), ranges=IRanges(start=start,end=end), strand=Rle(strand), marker=marker,pval=real1)

plotGrandLinear(gr, coord="genome", geom="line",size=0.3, aes(y=pval, group=seqnames), cutoff=c(def.thershold, def.thershold3), color="black", cutoff.color=c("blue", "green"), cutoff.size=0.3, space.skip=0.05, ylab="-log10(p.val)", main=QTL[1,i])+annotate("text", label=paste("thershold",def.thershold), y=def.thershold+2, x=as.numeric(1), size=3)+ylim(c(0,max(c(max(real1), def.thershold))+2))

ggsave(filename=paste(path, QTL[1,i], "half.pdf", sep=""))

data.frame(cbind(t(qtl[c(1:2),-c(1:21)]), paste("M", seq(1:length(start))),-log10(real)))->results

write.table(results, file=paste(path,qtl[1,i],"half.csv", sep=""),sep=";", na="NA", dec=".", row.names=F, col.names=T)

## 4. SCRIPT USED TO MODEL GROWTH AND KINETIC DATA.

The file to start should be adapted to the follwoing disposition. Each line of the excell file should correspond to one sample, each column, to one time point. If informative data wants to be added, it should be written in the first columns.

path<-"/Users/maria/Desktop/Rdir/"

read.csv(paste(path,"pressure.csv", sep=""), header = FALSE, sep = ";",dec=".",na.strings="NA")->data

dim(data)[1]->nl

temp<-(as.numeric(as.character(as.matrix(data[1,]))))[-c(1:3)]

data[-c(1),]->data

for (i in 4:nc){

as.numeric(as.character(data[,i]))->data[,i]

}

levels(data[,1])->LV

layout(matrix(c(1:16),4,4,byrow=TRUE))

for (i in 2:length(LV)){

which(data[,1]==LV[i])->s294_A

rainbow(length(s294_A))->clr

plot(temp,data[s294_A[1],-c(1:3)],type='n',main=c("strain",LV[i]),xlab="time",ylab="Pressure",ylim=c(0,max(data[,4:dim(data)[2]])))

for (j in 1:length(s294_A)){

points(temp,data[s294_A[j],-c(1:3)],col=clr[j])

}

}

# Logistic Fit ################################

data[,1:15]->PAR

PAR[,4:15]<-NA

colnames(PAR)[4:15]<-c("K","r","m","ip","t0.5","t1","t2", "t3","t4","t5","t5.5","tK")

round(dim(data)[1]/15,0)+1->nbg

library(plyr)

xvalue<-seq(from=0, to=max(temp), by=2)

####5PL

logistic5pl=function(x,y){

† Cmax0<-max(y)

† Cmin0<-min(y)+0.1

† T50<-as.numeric(x)[((y-max(y)/2)^2)==min((y-max(y)/2)^2)]

† r0<-log(Cmax0/Cmin0-1)/T50

† ip0<-T50/5

† fivepl<-nls(y~Cmax-(Cmin0+((Cmax-Cmin0)*(((exp(ip-(r*as.numeric(x)))))/(1+exp(ip-(r*as.numeric(x)))))^m)),start=list(Cmax=Cmax0,r=r0,m=0.8,ip=† ip0),control=nls.control(maxiter=300000,warnOnly=FALSE,minFactor = 1/32768),trace=T,algo="port",lower=c(3.5,0,0,0), upper=c(8,1.5,2,6),)

summary(fivepl)$parameters[1,1]->Cmax3 ; summary(fivepl)$parameters[2,1]->r3 ; summary(fivepl)$parameters[3,1]->m3 ; summary(fivepl)$parameters[4,1]->ip3

†yfivepl<-Cmax3-(Cmin0+((Cmax3-Cmin0)*(((exp(ip3-(r3*xvalue))))/(1+exp(ip3-(r3*xvalue))))^m3))

list(yfivepl=yfivepl,K=(summary(fivepl)$parameters[1,1]),r=(summary(fivepl)$parameters[2,1]),m=summary(fivepl)$parameters[3,1],ip=summary(fivepl)$parameters[4,1])

}

k<-1

for (nn in 1:nbg){

graphics.off() ; dev.new(50,30) ;

layout(matrix(c(1:15),3,5)) ; par(mar=c(2.5,2.5,0.5,0.5),mgp=c(1.5,0.6,0))

for (i in 1:15){

if (k<(dim(data)[1]+1)){

plot(temp,data[k,-c(1:3)],type="p",pch=20,cex=0.7,main=paste(data[k,1],k, sep=""),xlab="Time (H)",ylab="pressure")

†

#ajustement sur 5PL (five paremeters log-logistic)

xx<-temp ; yy<-as.numeric(data[k,-c(1:3)])

safe5pl<-failwith(NA,logistic5pl,quiet=TRUE)

yfivepl<-safe5pl(xx,yy)

if (is.na(yfivepl)==FALSE) {

points(xvalue,yfivepl$yfivepl,col="blue",type="l")

text(max(xx)-((max(xx)-min(xx))/3),min(yy)+((max(yy)-min(yy))*0.4),"5PL",col="blue",bty="n")

PAR[k,"K"]<-yfivepl$K

PAR[k,"r"]<-yfivepl$r

PAR[k,"m"]<-yfivepl$m

PAR[k,"ip"]<-yfivepl$ip

min(which(yfivepl$yfivepl>0.5))->t0

PAR[k,"t0.5"]<-xvalue[t0]

min(which(yfivepl$yfivepl>1))->t1

PAR[k,"t1"]<-xvalue[t1]

min(which(yfivepl$yfivepl>2))->t2

PAR[k,"t2"]<-xvalue[t2]

min(which(yfivepl$yfivepl>3))->t3

PAR[k,"t3"]<-xvalue[t3]

min(which(yfivepl$yfivepl>4))->t4

PAR[k,"t4"]<-xvalue[t4]

min(which(yfivepl$yfivepl>5))->t5

PAR[k,"t5"]<-xvalue[t5]

min(which(yfivepl$yfivepl>5.5))->t5.5

PAR[k,"t5.5"]<-xvalue[t5.5]

min(which(yfivepl$yfivepl>0.95*yfivepl$K))->tK

PAR[k,"tK"]<-xvalue[tK]

text(max(xx)-((max(xx)-min(xx))/3),min(yy)+((max(yy)-min(yy))*0.4),"no 5PL",col="blue",bty="n")

}}

k<-k+1

}

dev.copy2pdf(file=paste(path,k,"2fal.pdf"),out.type="pdf")

}

write.table(PAR, file =paste(path,"secondFAL.csv",sep=""), sep = ";", na = "NA", dec = ".", row.names = F, col.names = T, append=FALSE)
