# Supplementary material for: Genetic Causes of Phenotypic Adaptation to the Second Fermentation of Sparkling Wines in Saccharomyces cerevisiae
Source: G3 (Bethesda). 2016 Nov 28;7(2):399–412. doi: 10.1534/g3.116.037283 (PMC5295589; doi:10.1534/g3.116.037283)
Supplement: Supplementary file 3 [file 399FileS2.docx]

# File S2 MOLECULAR THECNIQUES

## 1. PCR TO AMPLIFY DELETION CASSETTES TO TRANSFORM

*KAN* cassette

≈500pb

≈500pb

- DNA extracted using Wizard genomic DNA purification kit (Promega).
- Primers used:

Table 1. Primers used to amplify each gene specific deletion cassette.

| primer identification | sequence | Tm | Gene deletion cassette |
| --- | --- | --- | --- |
| P1071 | AGAAGAAAGACCATGGAGTG | 55.3 | *SRO7* fw - |
| P1072 | TGGACTGGTTATTTACCCGA | 55.3 | *SRO7* rev |
| P1074 | CTCACGATCAAAAACAGGCA | 55.3 | *VMA13* fw - |
| P1075 | \|  \| ATGACTAACCGCCAGTCAC \| \| --- \| --- \| | 56.7 | *VMA13* rev - |
| P1086 | CTGCTTATTTCGAGTTCAC | 52.4 | *GSC2* fw |
| P1087 | CCACGTTGAATCCAAAAAGA | 53.2 | *GSC2* rev |
| P1092 | ATATCTGTCCAACACAACAGGT | 56.5 | *MSP1* fw |
| P1093 | TGTGGTGGATTATCCGTCAT | 55.3 | *MSP1* rev |
| P1095 | AAAGGCTCGCTCTGAAACA | 54.5 | *MSB2* fw |
| P1096 | GAACCTGTTGGATGAAGTG | 54.5 | *MSB2* rev |
| P1098 | TTAACAGTGGAGGAACAGTG | 55.3 | *SEC9* fw |
| P1099 | CCCTGTTTTCGAAGGAGTTT | 55.3 | *SEC9* rev |
| P1101 | CCTACGTTATTACCACTGTTTGG | 58.9 | *PDR1* fw |
| P1102 | GCCCCATAAGAAATACACCTCA | 58.4 | *PDR1* rev |
| P1104 | TCCTATAACACCAATAGTGAAAATC | 56.4 | *PMA1* fw |
| P1105 | GTTGAAATGTGCGTGTTGTG | 55.3 | *PMA1* rev |

- PCR mix:
  - 4ul Taq&GO,
  - 0.5ul primer forward,
  - 0.5ul primer reverse
  - 14ul sterile H_2_O,
  - 1ul DNA template.
- PCR program:

PCR conditions to amplify deletion cassettes:

- *SRO7*, *VMA13, MSP1, MSB2, SEC9, PMA1*

| 95º 5’ | **35x** | 72º 10’ | 10º infinte |
| --- | --- | --- | --- |
|  | 95º 30’’ |  |  |
|  | 54.5º 35’’ |  |  |
|  | 72º 2.30’ |  |  |

- *GSC2*

| 95º 5’ | **35x** | 72º 10’ | 10º infinte |
| --- | --- | --- | --- |
|  | 95º 30’’ |  |  |
|  | 52.5º 35’’ |  |  |
|  | 72º 2.30’ |  |  |

- *PDR1*

| 95º 5’ | **35x** | 72º 10’ | 10º infinte |
| --- | --- | --- | --- |
|  | 95º 30’’ |  |  |
|  | 57º 35’’ |  |  |
|  | 72º 2.30’ |  |  |

- Verification correct amplification on agarose gel 1%

## 2. TRANSFORMATION PROTOCOL

Based on the Gietz 2007 protocol. For more information:

<http://home.cc.umanitoba.ca/~gietz/>

PROCEDURE:

1.- The previous day (16h):

Inoculate the needed volume of YPD at an initial OD of 0.001.

The volume needed will depend on the number of transformations to perform.

2.- The day to perform the transformation:

- Wait for OD to reach between 0.6 – 1. Write down OD.
- Wash with water. (3000G 5minuts @ 4º)
- Wash with TE/LiAc (3000G 5minuts @ 4º)
- Eliminate supernatant. Dilute pellet to have a cellular concentration of 2.10^9^ cel/ml (1OD=2.10^7^cel/ml)
- Incubation 15 minutes at 30º without agitation.
- While incubation:
  - Prepare mix transformation for as many samples as transformations + negative control +1:
    - 240 ul of PEG
    - 36ul of LiAc
    - 50 ul of DNA carrier
    - 3.6 ul DMSO
  - Distribute 326 ul of mix in eppendorfs.
  - Add 34 ul of DNA to transform.
  - Vortex. Vortex. Vortex.
- At the end of incubation time, add to each eppendorf (remember negative control) 50 ul of cellular suspension. Mix by pipetting gently.
- Incubation 30 minutes at 30º without agitation
- Shock thermic 20 minutes at 42º.
- Quick centrifugation to pellet cells ( 30’’ , max speed)
- Discard supernatant. Dissolve pellet in 1 ml of YPD. Incubation minimum of 4h at 30º
- Spread yeast with digralsky spatula (very gently!!!) in YPD+ antibiotic (or other selection). Incubation at 30º during minimum 2-4 days.

3.- If after 2-4 days there are colonies. Sub-culture them in YPD+ antibiotic to confirm the positive.

MEDIA TO PREPARE.

1. DNA carrier (salmon sperm)

Adjust initial stock concentration to 2mg/ml. Dilution using TE.

Aliquots of 100ul in PCR tubes

Denaturalize at 95º during 5’, put in ice. Repeat 3 times.

1. TE 10x

Tris-HCl (pH=7.5) 1M = 5ml

EDTA 0.5M= 1ml

H2O to 50 ml

Or all previous solution are already steril or needed to be filtrated

1. LiAc ( Acetate de litium) 10x

10x == 1M

0.51g in 5 ml of H2O

Filtrate

1. PEG 50%

25g de PEG (3350 / 3500) dissolved in 25ml H2O.

Needs to be heated to dissolve.

Needs to be autoclaved.

**Needs to be fresh.** Preparation the day before transformation.

1. TE/LiAc

TE10x 10ml

LiAC 10x 10ml

H2O to 100ml.

- *DNA quantity to use: between* ***3-5 ug****. Above 10ug seems to be too much, recover of plenty of ectopic transformants.*
- *Time of recover after transformation. Important to wait* ***at least*** *4h.*
- *When plating the cells yeast after the recover time (last step) be very gently.*
- *The heat shock time may vary between 40-10 minutes. I have never obtained good results when applying the thermic shock during 40 minutes. Per contra, 10 minutes seemed enough to obtain transformants. The number of transformants obtained increases steadily from 10 to 20 minutes of shock thermic.*

## 3. PCR TO VERIFY TRANSFORMATION

Before transformation:

Gene X

After transformation

*KAN* cassette

p reverse:

**p560 bis**

p forward

≈400pb

The PCR verification consist in using one primer at 400pb (approx.) from the deleted gene loci and one primer that will anneal inside the *KAN* cassette. Thus, we verify the correct insertion of the KAN cassette in the right loci.

- PCR performed directly from colony
- PCR mix:
  - 4ul Taq&GO,
  - 0.2 primer forward,
  - 0.2 primer reverse,
  - 15.6 H2O.
- Distribute it in PCR tubes. Maintain in ice.
- In each tube, dissolve very little quantity of colony.
- PCR program:

| 95º 10’ | **35x** | 72º 5’ | 10º infinte |
| --- | --- | --- | --- |
|  | 95º 45’’ |  |  |
|  | 54º 45’’ |  |  |
|  | 72º 2’ |  |  |

- Primers used:

Table 2. Primers used in the verification of the transformation.

| Primer id | sequence | Tm | Gene |
| --- | --- | --- | --- |
| P560bis | 5’CGGCGCAGGAACACTG3’ | 56.9 | *KAN* test insertion  Anneals at the middel of the *KAN* cassette. |
| P1073 | CATCAAAGGCAAAAGAGCAAG | 55.9 | *SRO7* test insertion |
| P1076 | ATGTGTGAAACTGTTTGCGGT | 55.9 | *VMA13* test insertion |
| P1088 | TCGAACATGGAATTGTGGCT | 55.3 | *GSC2* test insertion |
| P1094 | \|  \| TCAATACAATTGGTCAAGGAAGTT \| \| --- \| --- \| | 55.9 | *MSP1* test insertion |
| P1097 | ACGGGGAAACGATAGCTGAT | 57.3 | *MSB2* test insertion |
| P1100 | TGTAGTTCTTCATTTGGCTGTG | 56.5 | *SEC9* test insertion |
| P1103 | CTGCAGCTTCTCACTATTATC | 55.9 | *PDR1* test insertion |
| P1106 | GTGACGAAACGTGGTCGA | 56.0 | *PMA1* test insertion |

- Verification in agarose gel 1%.

## 4. RFLP

RFLP TO CHECK IF THE REMAINING ALLELE IN THE TRANSFORMANT IS SB OR GN.

1. PCR to amplifly fragment that will be digested.

PCR mix:

4ul Taq&GO,

0.2 primer forward ,

0.2 primer reverse 0.2,

1ul genomic DNA (DNA extracted using Wizard genomic DNA purification kit (Promega),

14.6 H2O.

Program:

| 95º 3’ | **35x** | 72º 3’ | 10º infinte |
| --- | --- | --- | --- |
|  | 95º 35’’ |  |  |
|  | **TM** 30’’ |  |  |
|  | 72º 40’’ |  |  |

| **Fragment to amplify:** | primers: | Tm (**X**) |
| --- | --- | --- |
| ***PDR1*** | P1141/1142 | **54º** |
| ***BMH2*** | P1145/1146 | **54º** |
| ***PMA1*** | P1125/1126 | **54º** |
| ***SEC9*** | P1133/1134 | **54º** |
| ***SRO7*** | P1127/1128 | **54º** |
| ***MSB2*** | P1139/1140 | **54º** |
| ***WHI4*** | P1143/1144 | **54º** |
| ***SEP7*** | P1131/1132 | **54º** |
| ***GSC2*** | P1135/1136 | **54º** |
| ***MSP1*** | P1137/1138 | **52º** |
| ***VMA13*** | P1129/1130 | **51º** |

Tm, depending of the gene to verify;

In the following table, the primers used are listed:

Table 3. Primers used in the RFLP

| P1125 | ATGGCTTGTTTCTTAGCCAAG | 55.9 | RFLP *PMA1* forward |
| --- | --- | --- | --- |
| P1126 | AAGGTTTCATGGTTGTTACCG | 55.9 | RFLP *PMA1* reverse |
| P1127 | TCAATAACCTCTTATGAAGGTATGA | 56.4 | RFLP *SRO7* forward |
| P1128 | GATAGATTCACTTCAAAGCTAGCA | 57.6 | RFLP *SRO7* reverse |
| P1129 | ATGGGCGCAACCAAAATT | 51.4 | RFLP *VMA13* forward |
| P1130 | TCTGCTCAATGTTTTGCAAG | 53.2 | RFLP *VMA13* reverse |
| P1133 | GGCTACGGAACTCTGTTTC | 56.7 | RFLP *SEC9* forward |
| P1134 | TGAACAGGTCTGCAAATCCA | 55.3 | RFLP *SEC9* reverse |
| P1135 | TTGGACAGGCAAATGGTACA | 55.3 | RFLP *GSC2* forward |
| P1136 | ACCAGGTCTTTAAGGATGGC | 57.3 | RFLP *GSC2* reverse |
| P1137 | CTCGCAAATTTGATTTAAAAACG | 53.5 | RFLP *MSP1* forward |
| P1138 | TAGGGCCTTCGCCAACAT | 56.0 | RFLP *MSP1* reverse |
| P1139 | TAGCACCAGTGCTCCAAGT | 56.7 | RFLP *MSB2* forward |
| P1140 | CCTCAGAACTGCCAGTACTAC | 57.3 | RFLP *MSB2* reverse |
| P1141 | GGCAATTGAGGCGATTCAAT | 55.3 | RFLP *PDR1* forward |
| P1142 | TCTCAGTGGCCGAATTCATT | 55.3 | RFLP *PDR1* reverse |

1. Digestion. Overnight. Quantities indicated for a final restriction volume of 20ul. Restrictions conducted in PCR tubes.

Table 4. Digestions enzymes, Tª , digestions mix for each gene.

| **Enzyme** | **Gene** | **DNA**  **PCR product** | **Buffer** | **BSA** | **Enzyme (2U)** | **H2O** | **Tª** |
| --- | --- | --- | --- | --- | --- | --- | --- |
| MnlI | *PMA1, MSB2* | 5 | 2 | 0.2 | 0.4 | 12.4 | 37º |
| Mae III | *SRO7* | 5 | 2 |  | 1 | 12 | 55º |
| TspRI | *SEC9* | 5 | 2 | 0.2 | 0.2 | 12.6 | 65º |
| NsiI | *GSC2* | 5 | 2 |  | 0.2 | 12.8 | 37º |
| RsaI | *PDR1* | 5 | 2 |  | 0.2 | 12.8 | 37º |
| HaeIII | *VMA13* | 5 | 2 |  | 0.2 | 12.8 | 37º |
| BsaBI | *MSP1* | 5 | 2 |  | 0.2 | 12.8 | 60º |

Verify results in gel agarose 2%. Always BN, SB and GN as controls and one negative control (without DNA).
