# Supplementary material for: Genetic Causes of Phenotypic Adaptation to the Second Fermentation of Sparkling Wines in Saccharomyces cerevisiae
Source: G3 (Bethesda). 2016 Nov 28;7(2):399–412. doi: 10.1534/g3.116.037283 (PMC5295589; doi:10.1534/g3.116.037283)
Supplement: Supplementary file 6 [file 399FileS4.docx]

File S4. List of 97 Pma1p protein sequences. (.txt, 78 KB)

[http://www.g3journal.org/lookup/suppl/doi:10.1534/g3.116.037283/-/DC1/FileS4.txt](http://www.g3journal.org/lookup/suppl/doi:10.1534/g3.116.037283/-/DC1/FileS3.txt)
