# Supplementary material for: Genetic Causes of Phenotypic Adaptation to the Second Fermentation of Sparkling Wines in Saccharomyces cerevisiae
Source: G3 (Bethesda). 2016 Nov 28;7(2):399–412. doi: 10.1534/g3.116.037283 (PMC5295589; doi:10.1534/g3.116.037283)
Supplement: Supplementary file 10 [file 399FileS6.docx]

File S6. List of 97 Pdr1 protein sequences. (.txt, 103 KB)

<http://www.g3journal.org/lookup/suppl/doi:10.1534/g3.116.037283/-/DC1/FileS6.txt>
