# Supplementary material for: Genetic Causes of Phenotypic Adaptation to the Second Fermentation of Sparkling Wines in Saccharomyces cerevisiae
Source: G3 (Bethesda). 2016 Nov 28;7(2):399–412. doi: 10.1534/g3.116.037283 (PMC5295589; doi:10.1534/g3.116.037283)
Supplement: Supplementary file 13 [file 399TableS1.docx]

Table S1. Genetic and phenotypic data used for the 117 progeny clones of hoBN. (.csv, 340 KB)

<http://www.g3journal.org/lookup/suppl/doi:10.1534/g3.116.037283/-/DC1/TableS1.csv>
