# Supplementary material for: Genetic Causes of Phenotypic Adaptation to the Second Fermentation of Sparkling Wines in Saccharomyces cerevisiae
Source: G3 (Bethesda). 2016 Nov 28;7(2):399–412. doi: 10.1534/g3.116.037283 (PMC5295589; doi:10.1534/g3.116.037283)
Supplement: Supplementary file 14 [file 399TableS2.docx]

Table S2. List of non-synonymous SNP found between SB and GN for the genes MSB2, PDR1, PMA1 and VMA13. (.xlsx, 10 KB)

<http://www.g3journal.org/lookup/suppl/doi:10.1534/g3.116.037283/-/DC1/TableS2.xlsx>
